# Population genomics reveals genetic structure and dispersal sources of the invasive herb *Solanum rostratum* in northern China

**DOI:** 10.3389/fpls.2026.1688630

**Published:** 2026-03-20

**Authors:** Lifen Hao, Yabin Liu, Qianmei Wu, Yuyu Li, Lizhu Guo, Hui Wang, Rui Wang, Kejian Lin

**Affiliations:** 1 Key Laboratory of Biohazard Monitoring and Green Prevention and Control for Artificial Grassland, Ministry of Agriculture and Rural Affairs, Hohhot, China; 2 Inner Mongolia Key Laboratory of Grassland Conservation Ecology, Grassland Research Institute, Chinese Academy of Agricultural Science, Hohhot, China; 3 State Key Laboratory for Biology of Plant Diseases and Insect Pests, Institute of Plant Protection, Chinese Academy of Agricultural Sciences, Beijing, China

**Keywords:** ddRAD-seq, grassland biosecurity, human-mediated dispersal, invasion genetics, *Solanum rostratum*

## Abstract

**Introduction:**

The invasion of *Solanum rostratum* is causing severe damage to grassland ecosystems in northern China, with the species now spreading rapidly through ongoing emergence of new populations. To prevent further expansion, it is urgent to understand its dispersal patterns and identify the sources of new invasions. This study tested two hypotheses: (i) new invasions originate from established populations that serve as anthropogenic dispersal hubs, and (ii) gene flow is asymmetric, reflecting patterns consistent with human-mediated jump dispersal.

**Methods:**

We integrated population genomics and invasion history by analyzing genome-wide single nucleotide polymorphisms (SNPs) from 261 individuals across 30 populations. Populations were categorized as early-invaded (long-established), mid-invaded (moderately recent), or newly invaded based on county-level invasion records. Phylogenetic relationships, population structure, gene flow patterns, and dispersal networks were reconstructed using phylogenetic, clustering, and gene flow analyses to trace evolutionary relationships and potential dispersal routes.

**Results:**

Phylogenetic analysis resolved seven major monophyletic groups (A–G), while population structure analysis inferred seven ancestral gene pools (P1–P7), with strong concordance between the two independent datasets. Early-invaded populations exhibited clear spatial genetic differentiation. In contrast, newly invaded populations showed genetic admixture with ancestries overlapping multiple gene pools, indicating multiple origins and supporting a mixed dispersal pattern involving both local spread and long-distance jump dispersal. Specifically, new invasions in the Xilin Gol (XL) grassland were traced to agricultural regions in Zhangjiakou (ZJ) and Chifeng (CF). Gene flow and network analyses identified these early-invaded agricultural regions as key dispersal hubs supplying migrants to newly invaded areas. Source populations exhibited higher genetic diversity (*Ne, Ho, He, Fis, π*), whereas newly invaded populations showed reduced diversity, consistent with founder effects.

**Discussion:**

These findings confirm the hypothesis of asymmetric, human-mediated jump dispersal and clarify the invasion genetic architecture of *S. rostratum*. By identifying agricultural regions as critical dispersal hubs, this study provides precise targets for early management interventions, offering actionable insights to prevent further expansion and enhance grassland biosecurity.

## 1 Introduction

In the context of globalization, the threat posed by invasive alien species has become increasingly severe. Biological invasions, largely driven by human activities, result in species spreading from their native ranges into new habitats (Falk-Petersen et al., 2006; Estoup and Guillemaud, 2010). The dispersal and proliferation of invasive species in non-native regions are key drivers of their ecological and economic impacts, making systematic investigation of invasion patterns crucial for effective management (Pyšek et al., 2020). However, accurately pinpointing invasion sources remains challenging, particularly for species with complex dispersal histories.

Population genomic analysis has emerged as a powerful solution to this challenge (Cristescu, 2015; McGaughran et al., 2024). This approach is predicated on the premise that the genetic changes experienced by invasive populations carry spatial and temporal signals of their dispersal history, enabling the reconstruction of invasion pathways by resolving fine-scale genetic structure, gene flow, and demographic history (Brown et al., 2022).

Upon establishing in new areas, invasive species often face environments starkly different from their original habitats, and their genetic trajectories are shaped by key mechanisms (Roura-Pascual et al., 2021). Introduced populations frequently undergo founder effects and genetic bottlenecks, which stochastically shift allele frequencies and can produce genotypes distinct from source populations (Sax et al., 2007; Dlugosch and Parker, 2008; Stern and Lee, 2020). Conversely, repeated introductions and gene flow can introduce novel genetic material, enhance diversity, and facilitate adaptation (Lavergne and Molofsky, 2007; Busch, 2011). The interplay of these processes, along with environmental filtering, determines whether populations develop divergent genetic structures that reveal origins or retain ancestral patterns indicative of pre-adaptation (Vandepitte et al., 2014; van Boheemen et al., 2017). A notable example is *Spartina alterniflora* in China, whose invasion success has been attributed to post-introduction admixture, forming a genetically distinct lineage (Bernik et al., 2016). Ultimately, these genetic signatures can be used to infer dispersal modes.

The dispersal of invasive populations typically involves both local, continuous movement and long-distance jumps, leading to genetic patterns ranging from isolation-by-distance to complex admixture (Ward et al., 2008; Benvenuto et al., 2012; Lee et al., 2024). When jump dispersal is mediated by human activities and follows a bridgehead dynamic—where established populations act as secondary sources—it should leave distinct genomic signatures (van Boheemen et al., 2017). Thus, in systems with documented human-mediated spread, genomic analyses can help decipher dispersal pathways and identify the relative roles of natural and anthropogenic drivers.

*Solanum rostratum* Dunal., an annual invasive herb native to Mexico and the USA, exemplifies such a system. Its spiny morphology harms livestock and humans, and its solanine alkaloids are neurotoxic to animals (Singh and Bagnall, 1968; Guo et al., 2011; Abu-Nassar and Matzrafi, 2021). The species’ high adaptability allows it to form unidominant communities, negatively impacting grassland biodiversity and ecological security (Bryson et al., 2012; Yang et al., 2019; Yu et al., 2021). First reported in China in 1981, it has since expanded to nine provinces, exhibiting a discontinuous and rapidly evolving distribution that necessitates a better understanding of its dispersal dynamics (Wang et al., 2018; Ozuzu et al., 2024).

Previous studies using microsatellite loci (SSRs) have explored the population structure of *S. rostratum* in China but were limited in resolving fine-scale spatial structure, quantifying nucleotide diversity, or detecting contemporary gene flow and admixture—all critical for tracing human-mediated dispersal (Zhao et al., 2013; Zhang and Lou, 2018; Olazcuaga et al., 2020; Trujillo-González et al., 2022). To overcome these limitations and specifically test hypotheses about ongoing, human-mediated jump dispersal and source-sink dynamics, we employed double-digest restriction site-associated DNA sequencing (ddRAD-seq) to generate genome-wide single nucleotide polymorphism (SNP) markers for 261 individuals from 30 populations across China. Based on its invasion history, we hypothesized that (i) newly invaded populations are genetically admixed and originate from multiple early-invaded populations that act as anthropogenic dispersal hubs (H1), and (ii) asymmetric gene flow reflects human-mediated dispersal rather than natural diffusion (H2). We used these data to estimate genetic structure, differentiation, gene flow, and diversity, aiming to trace invasion sources and dispersal pathways, thereby informing strategies to enhance grassland biosecurity.

## 2 Materials and methods

To test the hypotheses that new invasions originate from established anthropogenic hubs (H1) and are driven by asymmetric, human-mediated gene flow (H2), we integrated field sampling, double-digest restriction site-associated DNA sequencing (ddRAD-seq), and population genomic analyses. The following subsections describe in sequence: (1) the spatio-temporal sampling design, (2) DNA extraction and sequencing library preparation, (3) single nucleotide polymorphism (SNP) calling and filtering, and (4) the analytical framework for inferring population structure, gene flow, and genetic diversity.

### 2.1 Sample collection

Since *S. rostratum* was first reported in China in 1981, it has expanded to approximately nine provinces. Field investigations revealed a spatially heterogeneous distribution, with densely occupied populations clustered in regions including Zhangjiakou (ZJ), Chifeng (CF), Chaoyang (CY), Jinzhou (JZ), Urumqi (UR), Tongliao (TL), and Hinggan League (HG). Newly invaded populations were identified in Xilingol League (XL), Baotou (BT), Turpan (TU), and Bayannur (BY).

Based on invasion timelines reconstructed from county-level records, populations were categorized into three chronological invasion stages: early-invaded populations (earliest invasions, 1981–2004; e.g., Chaoyang first reported in 1981), mid-invaded populations (moderately recent, 2005–2014), and newly invaded populations (recently, 2015–2022). This temporal categorization, based solely on historical records, provides an independent framework against which genetic patterns indicative of dispersal sources (e.g., early-invaded populations as hubs) can be rigorously tested.

**Figure 1:**
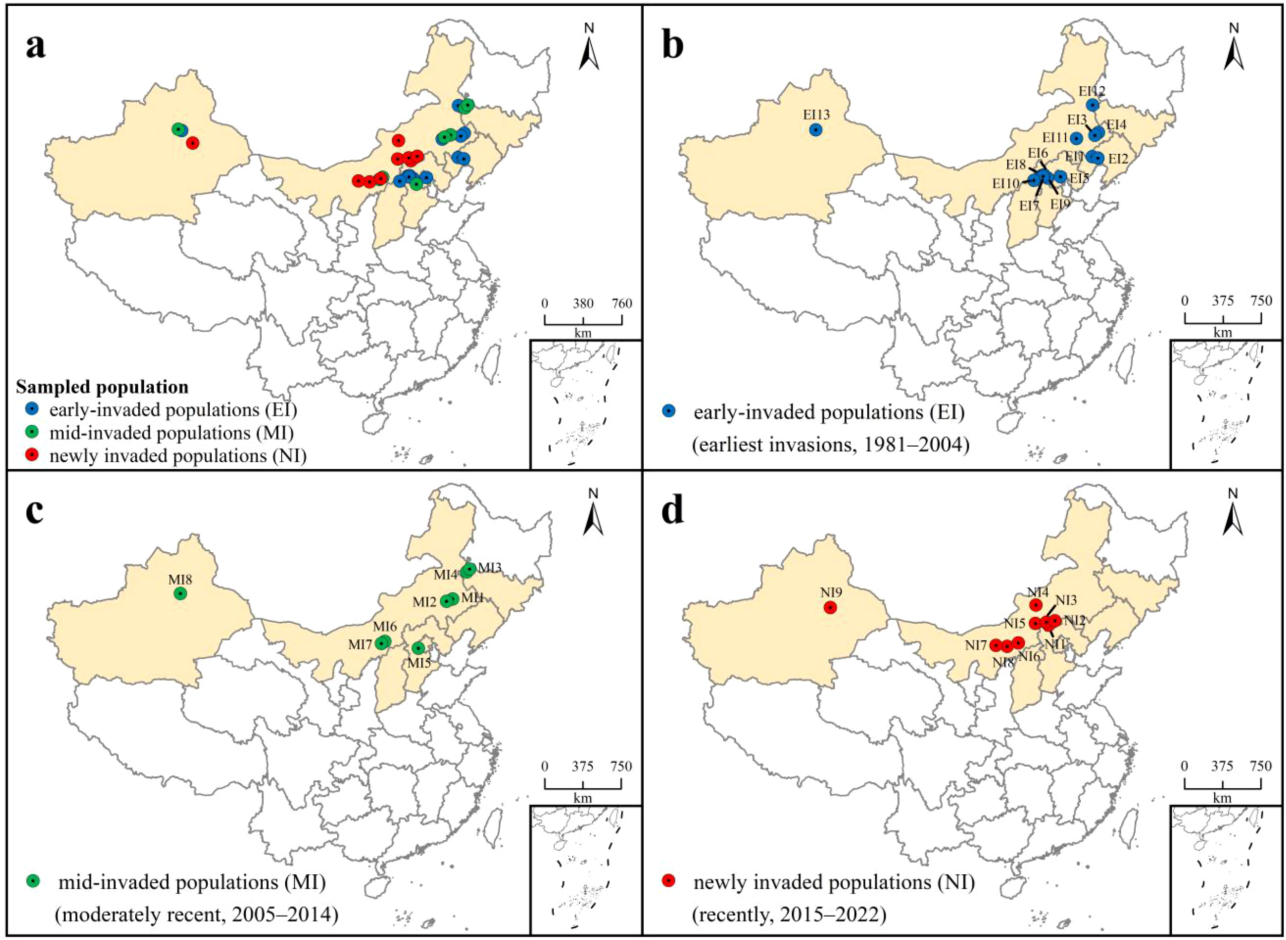
Study areas and sampling localities of *Solanum rostratum* in China. **(a)** Species distribution across China; **(b)** early-invaded (EI) populations (blue): EI1: CYBP, EI2: JZYX, EI3: TLKE, EI4: TLKL, EI5: BJMY, EI6: ZJQD, EI7: ZJXH, EI8: ZJHA, EI9: ZJHL, EI10: DTYG, EI11: CFBY, EI12: HGWL, EI13: XJUR; **(c)** mid-invaded (MI) populations (green): MI1: CFBZ, MI2: CFAL, MI3: BCZG, MI4: BCTB, MI5: BJMT, MI6: HHTZ, MI7: HHSH, MI8: XJCJ; **(d)** newly invaded (NI) populations (red): NI1: XLTP, NI2: XLZL, NI3: XLZB, NI4: XLSZ, NI5: XLXH, NI6: HHYQ, NI7: BYWQ, NI8: BTTY, NI9: XJTU.

Samples were collected from the typical habitats where *S. rostratum* occurs, including roadsides, grasslands, and the edges of agricultural fields. From July 2021 to August 2023, samples were collected according to the spatial distribution described above. A minimum of 6–10 plants per population was collected, with at least 5 meters between individuals to ensure genetic independence. Fresh leaves from each plant were stored in silica gel-packed plastic bags to preserve integrity. Precise geographical coordinates were recorded using a handheld GPS unit (Model UG908). In total, 261 individuals from 30 populations were collected (**Figure 1**; **Supplementary Table 1**). Population names were abbreviated using the first two capitalized letters of the Chinese names (in Pinyin) of their respective cities and counties. For example, samples from Xuanhua County, Zhangjiakou City, were assigned the abbreviation ZJXH, combining the city’s abbreviation (ZJ) and the county’s abbreviation (XH).

### 2.2 DNA extraction and sequencing

Genomic DNA was extracted from leaf tissues using the EasyPure Plant Genomic DNA Kit (Beijing TransGen Biotech Co., Ltd., Beijing, China), following the manufacturer’s protocol. DNA integrity was assessed via 0.8% agarose gel electrophoresis, while concentration and purity were measured using a Qubit fluorometer (Thermo Fischer Scientific). The extracted DNA was stored at -20 °C for subsequent analysis. We employed a double-digest restriction site-associated DNA sequencing (ddRAD-seq) approach for library preparation. For each sample, 200 ng of genomic DNA was double-digested with the restriction enzymes EcoRI (G^AATTC) and NlaIII (CATG^) (New England Biolabs, Beverly, MA, USA). The digested DNA was ligated to P1 and P2 adapters using T4 DNA ligase, followed by purification of ligated fragments using a fully automated magnetic bead system. Purified fragments were amplified via PCR, pooled, and re-purified.

Libraries with insert sizes of 300–500 bp were constructed using the ddRAD-seq method. Sequencing was performed on the Illumina Hiseq PE150 platform (Nanjing Jisihuiyuan Biotech, Nanjing, China), generating paired-end reads. Raw reads were filtered to remove contaminants, yielding clean reads for each sample. Clean reads were aligned to the *S. rostratum* reference genome (Zhang et al., 2023) using BWA v2.3.2 (http://bowtie-bio.sourceforge.net/index.shtml).

### 2.3 SNP calling and pipeline justification

For variant calling from the aligned dd-RAD sequencing data, we employed the Genome Analysis Toolkit (GATK v4.1.4.1). While alternative pipelines such as Stacks are commonly used for reduced-representation genomic data, the choice of GATK was justified by several study-specific factors: (1) the availability of a high-quality chromosome-level reference genome for *S. rostratum*; (2) an exceptionally high average mapping rate of 99.94% across all samples, which meets the prerequisite for reference-based variant callers; and (3) GATK’s robust capacity for applying stringent, site-level quality filters (e.g., QD, MQ, FS) to minimize false positives, which is crucial for downstream population genetic analyses. To confirm that our core conclusions were not dependent on the choice of SNP caller, we performed a parallel analysis using Stacks v2.3 (see Results section and **Supplementary Material**). The high concordance between the two pipelines validates the reliability of the GATK-based SNPs for this study.

The GATK pipeline was implemented as follows: Initial SNP sites were statistically analyzed, followed by recalibration and filtering with strict thresholds: QD < 2.0, MQ < 40.0, FS > 60.0, SOR > 6.0, MQRankSum < -12.5, and ReadPosRankSum < -8.0. Population-level SNP filtering was performed using VCFtools v0.1.16 with criteria including average sequencing depth >3, minor allele frequency (MAF) ≥0.05, SNP integrity >0.7, and allele count ≥2.

### 2.4 Data analysis

The genetic data were analyzed to (1) infer population genetic structure, phylogenetic relationships, and genetic differentiation (*Fst*); (2) investigate gene flow among populations; and (3) assess genetic diversity indices, including the number of valid alleles (*Ne*), observed heterozygosity (*Ho*), expected heterozygosity (*He*), inbreeding coefficients (*Fis*), and nucleotide diversity (*π*). This hierarchical framework—first identifying genetic clusters, then quantifying dispersal between them, and finally assessing their genetic variability—aligns with established practices in invasion genetics to trace invasion sources and dispersal mechanisms (Pyšek et al., 2020). The chronologically defined population stages (early-invaded, mid-invaded, and newly invaded) were used as a key grouping factor for comparative analyses of genetic diversity and to contextualize patterns of gene flow. Specifically, genetic structure and admixture analyses were used to test H1 (multiple origins of new invasions), while directional gene flow and network analyses were applied to test H2 (asymmetric, human-mediated dispersal).

#### 2.4.1 Population structure and phylogenetic analysis

We explored genetic structure by analyzing allelic data from ddRAD-seq across 261 samples (30 populations). Genetic ancestry was estimated using ADMIXTURE v1.3.0, which applies a maximum-likelihood approach to infer the optimal number of genetic clusters (*K*) and ancestry proportions. We ran 10 iterations for *K* values ranging from 2 to 20, selecting the best-fit model via cross-validation, and CLUMPP was also used for label swapping verification. Ancestry assignment probabilities were visualized using the R package *ggplot2*. To infer phylogenetic relationships, a Maximum Likelihood (ML) tree was constructed with FastTree v2.1.9 and visualized using FigTree v1.4.4. Fixation indices (*Fst*) and Tajima’s D statistics (with associated P-values from 10,000 neutral coalescent simulations, significance at *p* < 0.05) were computed using Arlequin v3.5.2.2. Geographic and genetic distance correlations were tested via Mantel tests in the R package *vegan*.

#### 2.4.2 Gene flow and genetic network

Gene flow among populations was quantified with BayesAss v3.0, which estimates the per-generation fraction of individuals in a population originating as migrants from elsewhere. We performed 10,000,000 bootstrap replicates with admixture parameter for allele frequency (-a) set to 0.25 and migration rate mixing parameter (-m) set to 0.05. Genetic connectivity was further analyzed using EDENetworks, a graph-theoretic method representing populations as nodes and genetic distances (*Fst*) as weighted edges. Edge thickness reflected connectivity strength, with a network threshold set at 0.32.

#### 2.4.3 Genetic diversity and differentiation analysis

Genetic diversity indices (*Ne*, *Ho*, *He*, *Fis*, *π*) were calculated using VCFtools v0.1.15. Nucleotide diversity (*π*) was specifically computed with a 100 kb sliding window (non-overlapping) across chromosomes. For regional analysis, *π* was estimated over a 10 kb window. Differences in genetic diversity indices (*Ne*, *Ho*, *He*, *Fis*, *π*) among the three invasion stages (early-invaded, mid-invaded, and newly invaded) were compared via one-way ANOVA at a 5% significance level.

#### 2.4.4 Validation of analytical robustness: parallel analysis with stacks pipeline

To assess the robustness of our population genetic inferences to the choice of SNP calling methodology, we conducted a parallel analysis using an independent pipeline. Raw reads were processed using Stacks v2.3 (Rochette et al., 2019), a software suite widely recommended for reduced-representation genomic data. The process_radtags module was used for demultiplexing and quality filtering. Loci were assembled and SNPs were called using the ustacks, cstacks, and sstacks modules with default parameters. Population-level filtering was applied with a minimum allele frequency (MAF) ≥ 0.05 and locus completeness > 70%. The resulting SNP dataset was then subjected to the same suite of downstream analyses (e.g., ADMIXTURE, phylogenetic tree construction, diversity estimation) as the GATK-derived dataset for direct comparison.

### 2.5 Field survey and documentation of a dispersal pathway

To empirically test the hypothesis of human-mediated long-distance jump dispersal suggested by the genetic patterns, we conducted a targeted field survey in October 2023. Focusing on the agricultural supply chain—a suspected vector due to the species’ common occurrence in farmlands—we inspected livestock feed sources at a herder’s residence in Zhenglan Banner, Xilingol League, a region of recent invasion. The survey successfully traced contaminated crop residues containing *S. rostratum* stems and seeds back to local forage markets and ultimately to their origin in crop fields of Zhangjiakou City, a known introduction hub. This directed survey was conducted to provide a concrete test for an anthropogenic dispersal pathway inferred from genomic patterns.

## 3 Results

### 3.1 Genome sequencing of *S. rostratum* in China

Using the ddRAD-seq approach described above, we generated and analyzed genome-wide data to test our hypotheses. Following sequence quality control, we obtained approximately 1.25 billion clean reads (amounting to 360.37 Gb of clean data) across all 261 samples. The average read count per sample was 4,794,134 (range: 3,194,543–7,624,751). Sequence quality was high, with an average Q30 base content of 92.11% and average GC content of 36.68%. An exceptional 99.94% of clean reads mapped to the *S. rostratum* reference genome. After stringent filtering, 3,827,290 high-quality SNPs were retained for downstream analyses (**Supplementary Table 2**).

### 3.2 Genetic population structure and phylogenetic relationships

**Figure 2:**
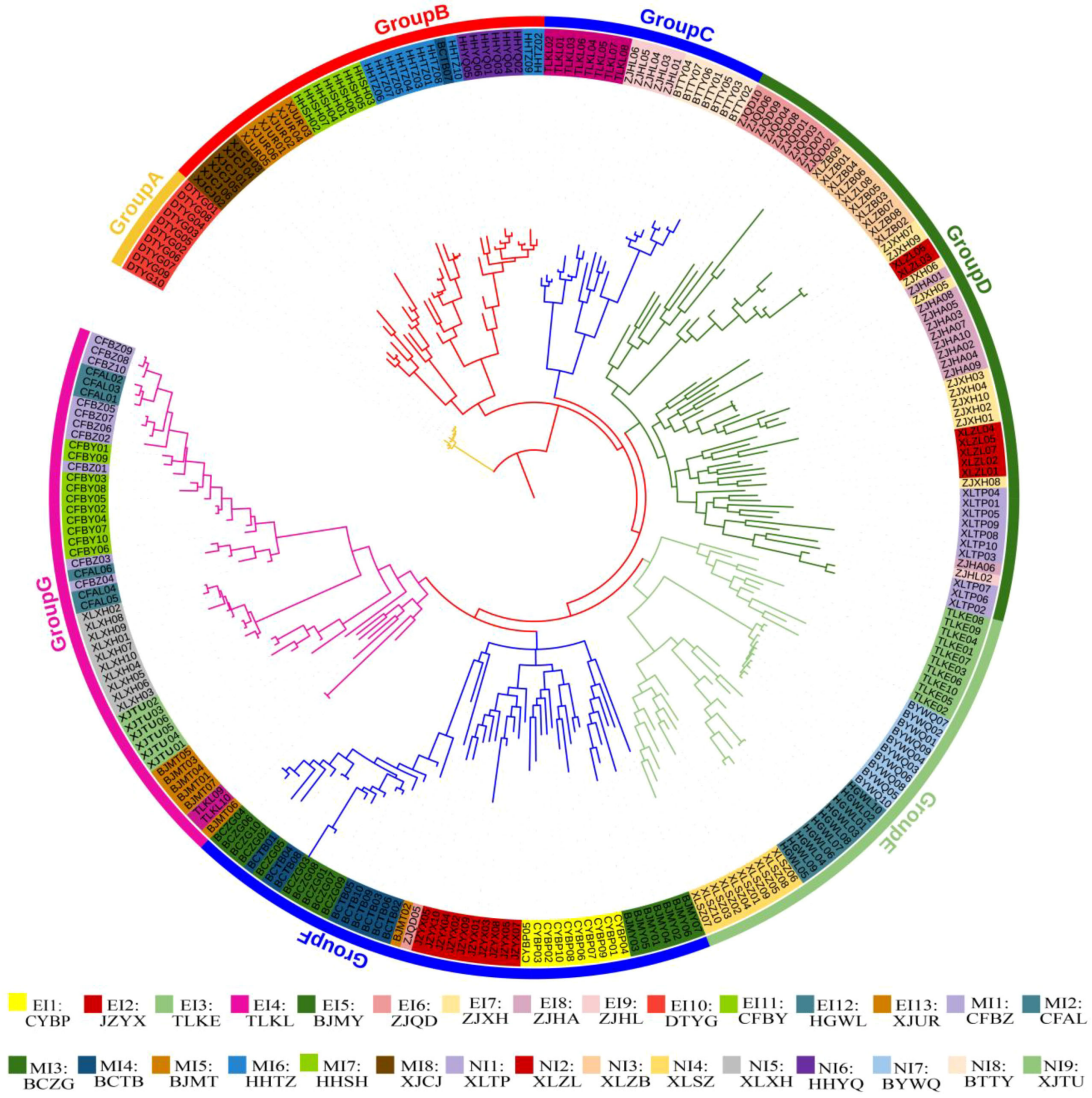
Maximum Likelihood phylogenetic tree of 261 *Solanum rostratum* individuals from 30 populations. The tree resolves seven major, monophyletic clades, labeled Group A–G. Each major clade is assigned a distinct background color. Within each clade, individuals are colored by their source population (see population codes in **Figure 1**), illustrating the genetic substructure and population composition of each genetic group.

Following the assessment of genomic data quality, we first examined the population phylogenetic tree to test whether newly invaded populations are closely related to their putative origins (H1). The Maximum Likelihood (ML) tree resolved the 261 individuals into seven major, monophyletic groups (Group A–G) (**Figure 2**). While most individuals clustered by their population of origin, two (BCTB07 and ZJQD05) were placed outside their primary population clusters. The tree revealed strong spatial genetic patterns. Key early-invaded populations—including DTYG (EI10) and XJUR (EI13)—each formed a distinct genetic cluster, as did populations identified as subsequent dispersal hubs, such as ZJXH (EI7) from Zhangjiakou and CYBP (EI1) from Chaoyang. This pattern suggests multiple independent introductions or early diversification. Notably, populations from the same city sometimes belonged to different genetic clusters (e.g., Beijing populations were divided between Group F and G), further underscoring the complexity of the invasion history.

**Figure 3:**
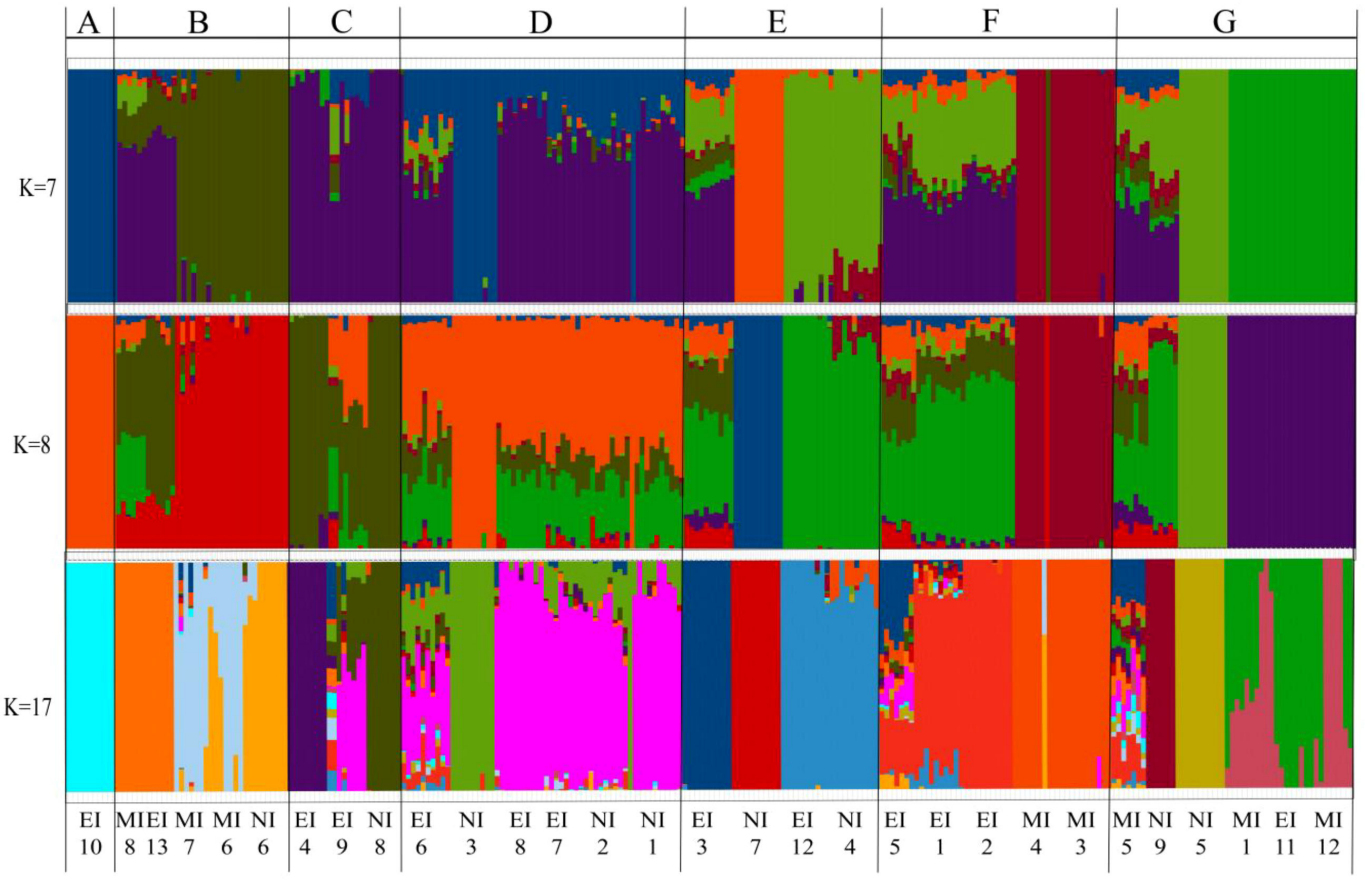
Genetic structure of *Solanum rostratum* populations inferred from ADMIXTURE analysis. Individual ancestry proportions (bar heights) are shown for three values of *K* (number of ancestral gene pools), including the optimal *K* = 17 (lowest cross-validation error), as well as the *K* = 7 and *K* = 8 configurations. At *K* = 7, each of the seven inferred gene pools (P1–P7) is represented by a distinct color (see color legend in **Figure 4**). The letters **(A–G)** above the *K* = 7 panel correspond to the seven major phylogenetic groups identified in the Maximum Likelihood tree (**Figure 2**).

The Maximum Likelihood phylogenetic tree and ADMIXTURE analysis largely converged in supporting the existence of seven distinct gene pools (*K* = 7, which was retained for subsequent ecological interpretation; **Figure 3**). A key finding supporting hypothesis H1 was that populations from newly invaded regions consistently exhibited genetic admixture, with ancestry components tracing back to multiple, divergent gene pools (**Figure 3**). For example, populations in the newly invaded Xilingol (XL) region showed ancestry profiles that overlapped with those of gene pools containing early-invaded populations from Zhangjiakou (ZJ) and Chaoyang (CY). Although the first two principal components of the Principal Component Analysis (PCA) accounted for only 14.32% of the total genetic variance, they clearly revealed the major patterns of population stratification. Specifically, the PCA showed that newly invaded populations clustered more closely with early-invaded populations than with mid-invaded ones (**Supplementary Figure 3**).

Overall, the patterns inferred from the phylogenetic tree, ADMIXTURE analyses (at *K* = 7, *K* = 8, and *K* = 17), and PCA were largely congruent. However, some populations (e.g., XLXH and Baicheng) showed inconsistent assignments between the Maximum Likelihood tree and the ADMIXTURE results at higher *K*-values (*K* = 7, K = 8, *K* = 17). More coherent signals regarding the deep ancestry of these populations emerged at a lower cluster number (*K* = 4) (**Supplementary Figure 5**), highlighting how different *K* values in ADMIXTURE capture different temporal depths of genetic structure.

Population differentiation (*Fst*) revealed strong geographic structuring (**Supplementary Table 3**). Mantel tests indicated a weak but significant isolation-by-distance pattern (*r* = 0.29, *p* = 0.03; **Supplementary Figure 2**; **Supplementary Table 4**). However, the pronounced genetic admixture observed in newly invaded populations stands in contrast to this weak pattern of isolation-by-distance. This spatial-genetic mismatch suggests that processes beyond natural diffusion, such as human-mediated jump dispersal, likely contributed to the formation of these new invasions.

**Figure 4:**
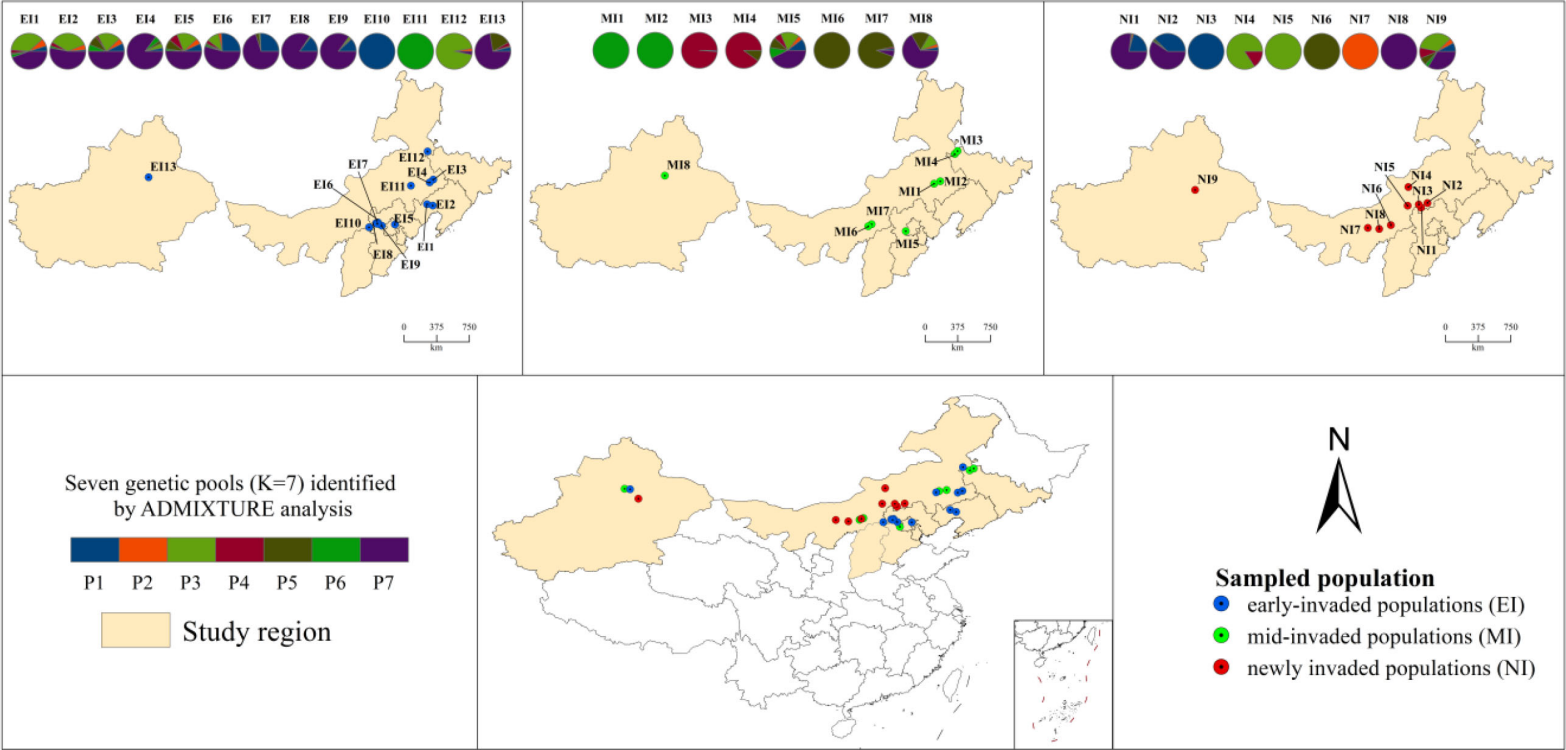
Spatial distribution of genetic ancestry across sampling locations. Pie charts display population-averaged ancestry proportions from the ADMIXTURE analysis at K = 7. Colors correspond to the seven gene pools defined in **Figure 3**. The area of each pie chart is proportional to the number of individuals sampled in that population. Sampled populations are marked as early-invaded (EI, blue circles), mid-invaded (MI, green circles), or newly invaded (NI, red circles). The mixed ancestry observed in newly invaded Xilingol (XL) populations suggests multiple origins; a pattern further investigated through gene flow analysis (**Figure 5**).

The spatial projection of the genetic structure (**Figure 4**) visualized this mismatch clearly. It showed that newly invaded regions, such as Xilingol League (XL), contained populations with admixed ancestries originating from multiple, geographically distant gene pools (e.g., those containing early-invaded sources from Zhangjiakou and Chaoyang). This spatial-genetic pattern indicates multiple origins for new invasions (H1).

### 3.3 Gene flow and genetic network

**Figure 5:**
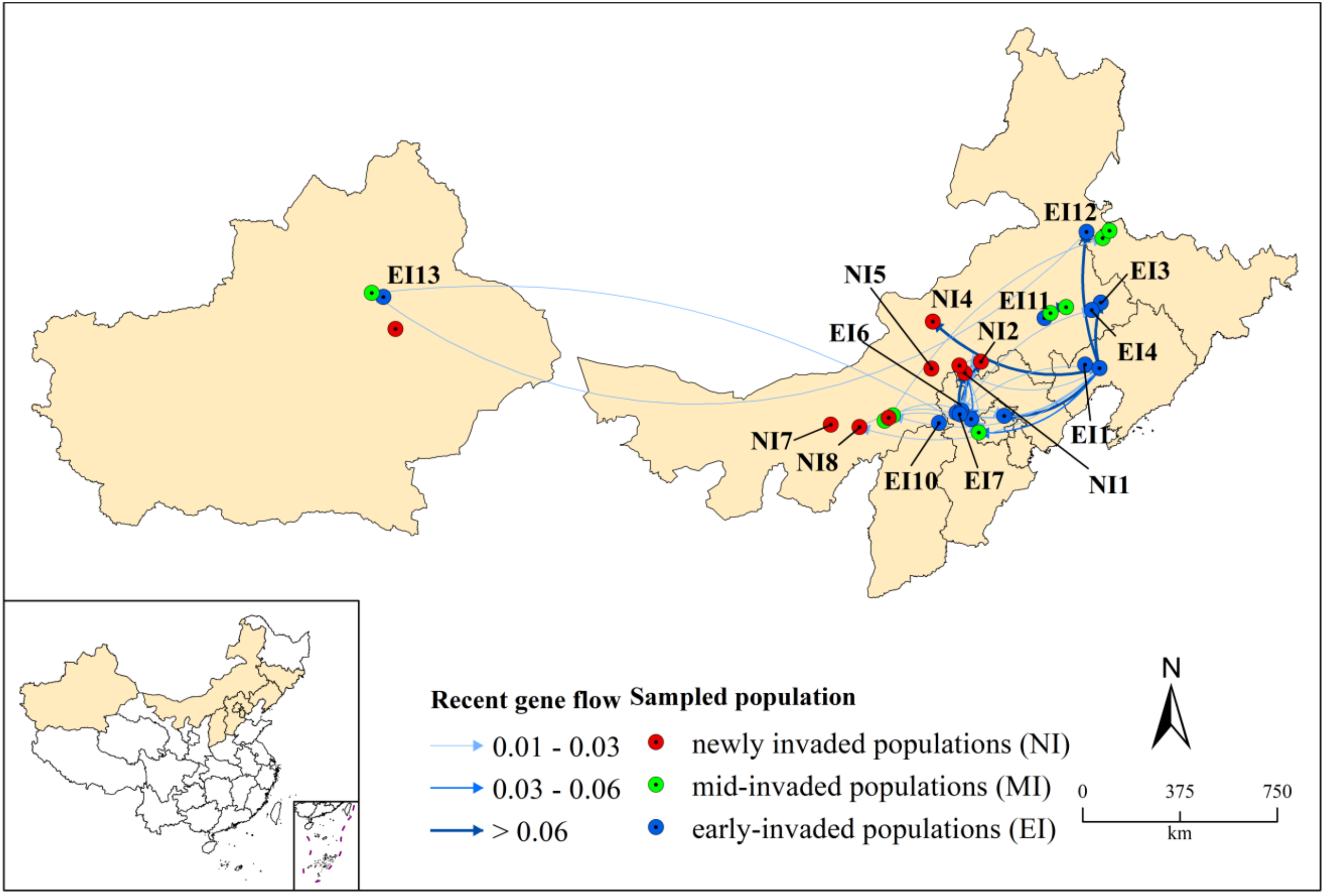
Asymmetric gene flow among *Solanum rostratum* populations. Arrows indicate the direction of gene flow (migration), with line color and thickness corresponding to the estimated migration rate (see **Supplementary Table 5**). Source populations identified by BayesAss analysis are labeled. Key source populations (e.g., ZJXH, CYBP) identified here correspond to central hubs in the genetic network (**Figure 6**).

To quantify dispersal directions and test for asymmetry (H2), we analyzed contemporary gene flow. Gene flow analysis revealed asymmetric dispersal dynamics (**Figure 5**, **Supplementary Table 5**). Populations from the early-invaded stage consistently contributed migrants to newly invaded regions. Bootstrap-supported results (*p* < 0.05) identified populations from Chaoyang (CYBP, EI1), Zhangjiakou (ZJXH, EI7), and Chifeng (CFBY, EI11/MI1) as primary contemporary sources. Specifically, gene flow was directed from these established hubs toward newly invaded populations in the Xilingol (XL) grasslands (e.g., XLTP, XLZL) and other recent frontiers (e.g., BTTY). For example, CYBP (EI1) showed significant migration to Baotou’s BTTY (migration rate = 0.008, *p* < 0.01). This pattern of asymmetric gene flow from early-invaded hubs to new fronts strongly supports H2 and is consistent with human-mediated jump dispersal.

**Figure 6:**
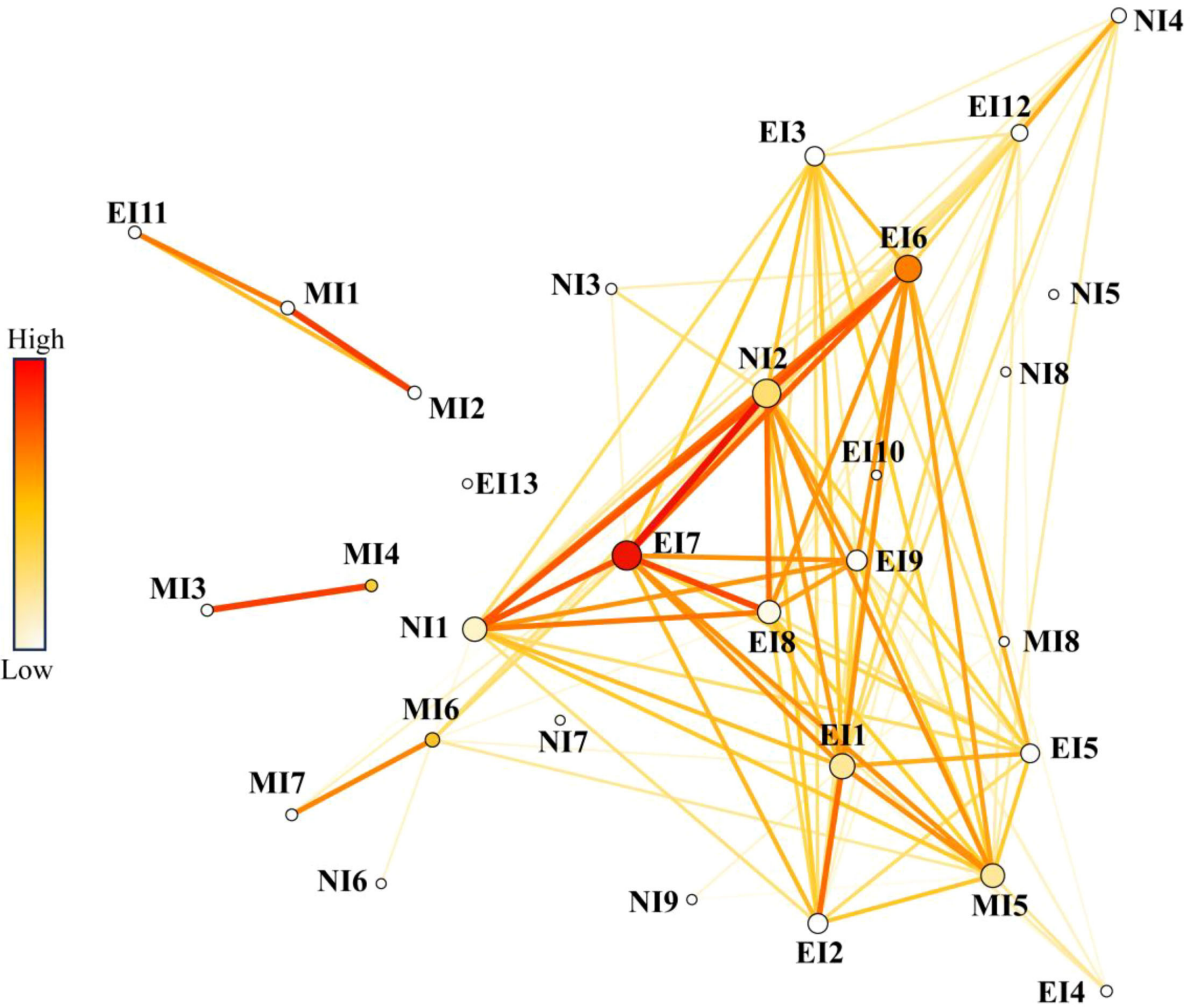
Genetic network of *Solanum rostratum* populations. Nodes represent populations, sized and colored by their cumulative weighted degree (a measure of connectivity). Edges represent pairwise genetic connectivity (1/*Fst*), with width and color intensity proportional to connection strength. The network is thresholded at *Fst* = 0.32. Populations central to the network (e.g., ZJXH, CYBP) were also identified as key dispersal hubs in the gene flow analysis (**Figure 5**). This concordance demonstrates that the topological hubs of the genetic network are the same populations acting as dispersal sources (**Figure 5**) and contributing genetic ancestry to new invasions (**Figures 2**, **4**).

The genetic network (threshold *Fst* < 0.32; **Figure 6**) resolved two broad geographic clusters but, more importantly, identified key hub populations based on topological centrality. Populations that functioned as major sources in the gene flow analysis—ZJXH (EI7), CYBP (EI1), and CFBY (EI11/MI1)—also occupied central positions in the genetic network, acting as nodes bridging different genetic groups and connecting to newly invaded areas. For instance, ZJXH (EI7) showed strong connectivity to newly invaded populations like XLZL (NI2) and XLTP (NI1). The concordance between the asymmetric gene flow sources (**Figure 5**) and the topological hubs in the genetic network (**Figure 6**) provides robust, multi-analytical evidence that these established populations are pivotal dispersal centers driving contemporary range expansion.

### 3.4 Genetic diversity across invasion stages

**Figure 7:**
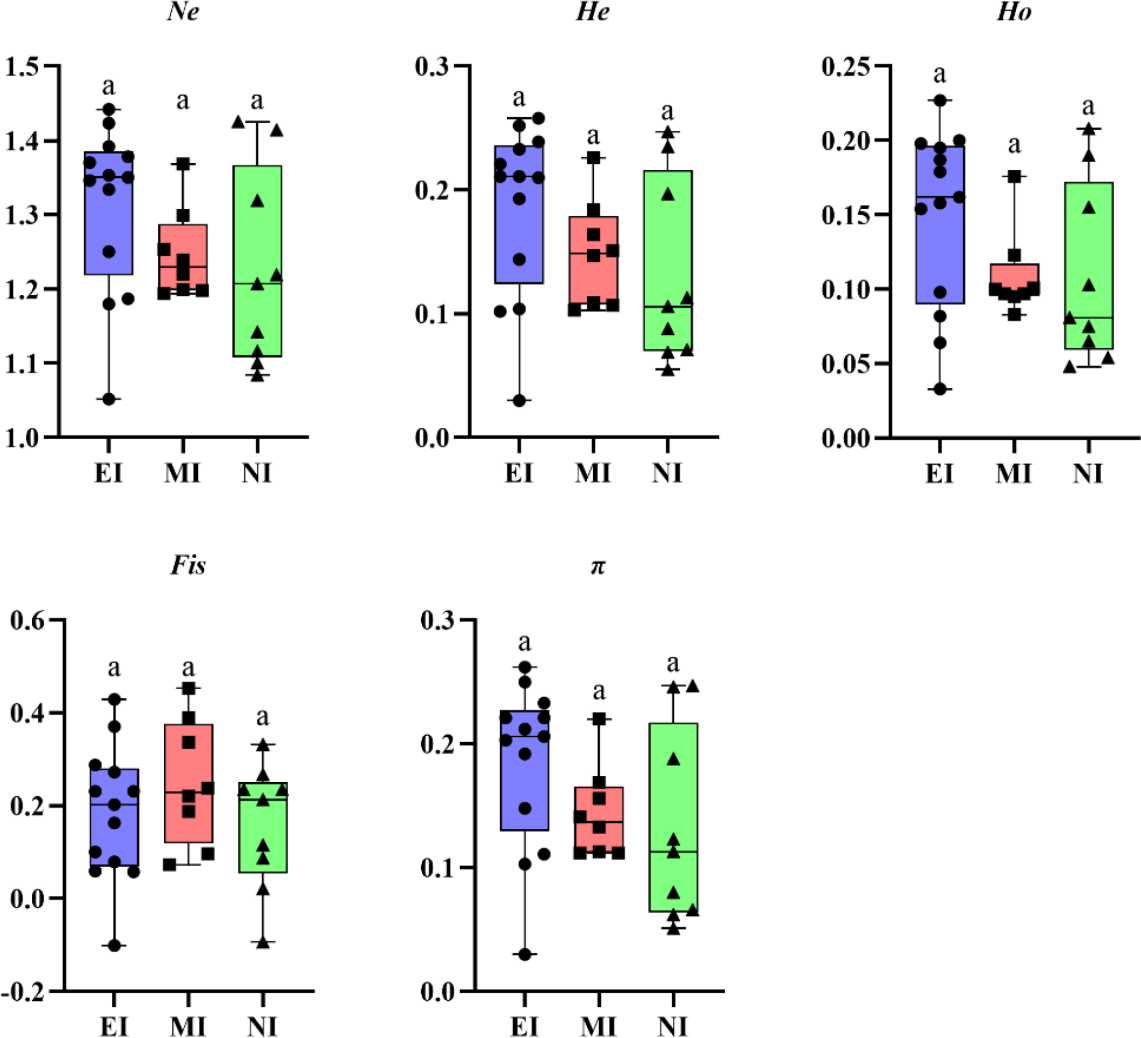
Comparative genetic diversity across three invasion stages. Boxplots show the distribution of the number of valid alleles (*Ne*), expected heterozygosity (*He*), observed heterozygosity (*Ho*), inbreeding coefficient (*Fis*), and nucleotide diversity (*π*) for early-invaded (EI), mid-invaded (MI), and newly invaded (NI) populations. Individual points represent the value for each population.

Given the identification of these dispersal hubs and asymmetric gene flow, we next asked whether populations at different invasion stages show differential genetic signatures. Although one-way ANOVA revealed no statistically significant differences (*p* > 0.05) in the overall comparison, the observed mean values of genetic diversity indices (*Ne, Ho, He, Fis*, π) showed a declining trend across the chronological invasion stages (**Figure 7**; **Supplementary Table 6**). Early-invaded populations that were identified as dispersal hubs (e.g., ZJXH, CYBP) retained the highest genetic diversity, whereas newly invaded populations (e.g., those in XL and BY) showed reductions in *Ne* and *π*, consistent with founder effects during colonization. For example, the early-invaded hub population ZJQD (EI6), located near Zhangjiakou, displayed the highest overall diversity (*π* = 0.262), while the newly invaded population BYWQ (NI7) showed markedly lower diversity (*π* = 0.051). This pattern indicates that high-diversity source hubs can spawn satellite populations that experience diversity loss, yet continue to drive invasion success through recurrent, human-mediated dispersal.

### 3.5 Documentation of a human-mediated dispersal pathway

To empirically investigate a potential anthropogenic vector for long-distance dispersal, a targeted field survey was conducted following the genetic analyses. In October 2023, at a newly invaded herder’s residence in Zhenglan Banner (Xilingol League), we identified dry stems and seeds of *S. rostratum* contaminating a batch of purchased livestock fodder. Tracking the supply chain of this fodder revealed a pathway: the material was obtained from a local forage market, which in turn sourced it from crop fields in Zhangjiakou City, Hebei Province. In Zhangjiakou, *S. rostratum* was confirmed as a common weed in the source croplands. This survey physically traced a complete route for the movement of *S. rostratum* propagules via the inter-regional trade of contaminated crop residues.

### 3.6 Concordance between SNP calling pipelines

Population genetic inferences based on the GATK SNP-calling pipeline were highly concordant with those derived from the independent Stacks v2.3 pipeline (see Methods 2.4.4). ADMIXTURE analysis on the Stacks-derived SNPs revealed nearly identical ancestry proportions and clustering patterns at *K* = 7 and *K* = 8. The maximum-likelihood phylogenetic trees constructed from each SNP set showed congruent topologies, strongly supporting the same seven monophyletic groups and the major gene pools at *K* = 7. Furthermore, the trends in all genetic diversity indices (*Ne*, *Ho*, *He*, *Fis*, and *π*) across invasion stages were qualitatively consistent between the two datasets. These comparative results confirm that the core biological conclusions of this study regarding population structure, phylogenetic relationships, and diversity patterns are robust to the choice of SNP calling methodology. Detailed comparative figures are provided in **Supplementary Figure 4**.

## 4 Discussion

Our integrated genomic analyses provide robust evidence that the spread of *S. rostratum* in China is characterized by genetic admixture in new invasions (supporting H1) and asymmetric, hub-driven dispersal (supporting H2). Building on these findings, we discuss the dual dispersal mechanisms, the role of established populations as sources, and the management implications for grassland biosecurity. This study employed double-digest RAD-seq (ddRAD-seq; Rochette et al., 2019) to generate genome-wide SNPs, enabling the resolution of fine-scale dispersal dynamics. By integrating genetic structure, gene flow networks, and invasion history (Pyšek et al., 2020), our work reveals a complex invasion architecture driven by anthropogenic dispersal pathways (Zhao and Lou, 2017; Rogers et al., 2019) operating alongside natural diffusion dynamics (Slatkin, 1993). This architecture fundamentally reshapes our understanding of dispersal routes, range expansion sources, and mechanisms for this ecologically damaging invader (Estoup and Guillemaud, 2010).

### 4.1 Genetic architecture reveals dual dispersal mechanisms

Our genetic structure analysis reveals that the spread of *S. rostratum* in China is not a unitary process, but a dual pattern. First, a weak but significant isolation-by-distance pattern (*r* = 0.29, *p* = 0.03) indicates that natural diffusion correlated with geographic distance has contributed to local range expansion, forming a baseline pattern consistent with gradual spread (Slatkin, 1993). However, the overall genetic structure deviates strongly from this simple isolation-by-distance expectation. Striking instances of genetic-geographic decoupling—such as high differentiation between adjacent populations (e.g., XLXH and XLZB, *Fst* = 0.705 despite only 107 km apart) and unexpectedly close genetic affinity between distant ones (e.g., BYWQ and TLKE, *Fst* = 0.522 across 1,127 km)—collectively provide robust genomic evidence for recurrent, long-distance jump dispersal (Wilson et al., 2009). Given the documented role of human activity in its spread, this pronounced decoupling prompted us to hypothesize a human-mediated mechanism for these jumps.

To investigate this, we conducted a hypothesis-driven field survey targeting the agricultural supply chain in the Xilingol region, a major sink indicated by gene flow analysis. This survey (Results 3.5) physically documented a complete anthropogenic dispersal route: *S. rostratum* propagules were transported via contaminated livestock fodder from agricultural fields in Zhangjiakou (a genetically identified source region) to a newly invaded pasture in Zhenglan Banner, Xilingol. This field evidence serves as a critical proof of existence for a specific vector—contaminated fodder trade—that directly translates the inferred genetic pattern of jump dispersal into an operational, real-world process (Beckman et al., 2019). While this single documented pathway may not account for all jump dispersal events, it decisively confirms that human-mediated transport is occurring and provides a concrete mechanism to explain the severe genetic-geographic decoupling we observed. This field evidence therefore concretely validates the human-mediated dispersal (H2) inferred from genomic patterns by confirming contaminated fodder trade as a vector for long-distance jump dispersal. The convergence of these independent lines of evidence—genomic patterns and field-documented vector—strongly supports our hypothesis that human-mediated dispersal, rather than natural diffusion alone, underpins the jump dispersal events in this system.

Therefore, by integrating fine-scale genetic structure from genome-wide SNPs analysis with field-based traceability evidence, this study demonstrates that the invasion of *S. rostratum* in China is a composite process shaped by both natural diffusion and human-mediated jump dispersal. This human-mediated jump dispersal, facilitated by agricultural activities, can effectively bypass geographic barriers and greatly accelerate the invasion process. This not only provides robust genomic support for previous inferences based on indirect evidence that human-mediated dispersal plays a key role in the spread of this species (Zhao and Lou, 2017; Rogers et al., 2019). But, more importantly, it directly links the genetically inferred dispersal pattern to a clear, specific anthropogenic pathway, thereby completing the full argument from pattern identification to mechanism confirmation.

Collectively, the decoupling of genetic patterns from geography, combined with direct field evidence of a specific anthropogenic vector (contaminated fodder), offers compelling support for our second hypothesis that human-mediated dispersal, rather than natural diffusion alone, underpins the jump dispersal events observed in this study. If human-mediated jump dispersal is prevalent, a critical question arises: which populations act as the primary sources for these dispersal events?

### 4.2 Established populations as dispersal hubs and invasion sources

Our genomic analyses pinpoint several established populations as pivotal contemporary dispersal hubs. The BayesAss analysis estimates migration rates over recent generations, which—given the known, recent invasion history of newly invaded populations—allows us to interpret significant gene flow as indicative of ongoing or very recent dispersal contributing to range expansion, rather than solely reflecting historical admixture.

Our results confirm both hypotheses: newly invaded populations are genetically admixed from multiple sources (supporting H1), and directional gene flow originates from specific early-invaded hubs (supporting H2). By integrating asymmetric gene flow (**Figure 5**), genetic network modeling (**Figure 6**), and genetic structure analyses (**Figures 2**, **3**), we identified key populations that function as these hubs. These included early-invaded populations in Chaoyang (CYBP, EI1), Tongliao (TLKL, EI4; TLKE, EI3), Zhangjiakou (ZJXH, EI7), and Chifeng (CFBY, EI11).

For instance, TLKL (EI4) supplied migrants to Baotou’s BTTY (migration rate = 0.008, *p* < 0.01), HGWL (EI12) contributed to the newly invaded population XLSZ (NI4), and the newly invaded BYWQ likely originated from TLKE (EI3). Zhangjiakou’s ZJXH (EI7) also acted as a bridge connecting disparate gene pools (**Supplementary Table 5**). Network analysis further confirmed these hubs as central nodes with high weighted connectivity (e.g., ZJXH linking to Xilingol’s XLZL (NI2) and XLTP (NI1) at *Fst* < 0.32 threshold). Crucially, these hubs represent strategic targets for “source-sink” interventions to disrupt invasion networks (Radosevich et al., 2003; Cristescu, 2015). This pattern—where populations established during early and mid-invasion phases function as genetic reservoirs and active dispersal hubs for more recent invasions—strongly supports a ‘bridgehead’ invasion dynamic within China, where established populations act as secondary sources for further spread.

Although our study did not include native-range populations for a formal comparative test, the dominant genetic connectivity and admixture among Chinese populations suggest that ongoing, direct introductions from the native range are not the primary driver of current expansion. Instead, a bridgehead dynamic mediated by established domestic hubs appears predominant. This interpretation is corroborated by invasion chronology: newly invaded populations in regions like Xilingol emerged years or decades after the establishment of these source hubs (e.g., Zhangjiakou, Chifeng and Hinggan), making direct, independent introductions from the native range a less parsimonious explanation. The role of these established populations as genetic reservoirs and dispersal hubs raises a further question regarding the genetic consequences of this bridgehead dynamic for both source and newly founded populations.

### 4.3 Resolving the genetic paradox through bridgehead dynamics

Consistent with their role as dispersal hubs, early-invaded populations retained the highest genetic diversity, while diversity declined in mid- and newly-invaded populations (**Figure 7**). For instance, early-invaded populations like ZJQD (EI6) exhibited the highest nucleotide diversity (π = 0.262), whereas newly invaded populations such as BYWQ (NI7) showed marked reductions (*π* = 0.051). This pattern reflects classic founder effects and bottlenecks during colonization (Dlugosch and Parker, 2008), with elevated inbreeding in isolated populations (e.g., XJUR (EI13): *Fis* = 0.370) further supporting demographic constraints. This presents an apparent “genetic paradox” of successful expansion despite diversity loss (van Boheemen et al., 2019; Cordero et al., 2023), which can be resolved by examining the bridgehead dynamic. The exceptional case of Zhangjiakou illustrates this: as a high-diversity hub (ZJQD (EI6): *Ne* = 1.442, *π* = 0.262), it spawned genetically diverse satellite populations in Xilingol grasslands. This demonstrates how multiple introductions from robust source populations can enhance invasive potential in new territories (Vandepitte et al., 2014; van Boheemen et al., 2017)—a key facet of the bridgehead effect. Conversely, the monophyletic, low-diversity DTYG (EI10) clustered group(*π* = 0.03) illustrates how single-introduction events can drive rapid local adaptation without genetic richness (Gioria et al., 2023).

### 4.4 Management implications for grassland biosecurity

The genetic insights from this study—specifically the identification of source hubs and the understanding of their diversity dynamics—directly inform targeted, preemptive management strategies for *S. rostratum*. Our genomic reconstruction identifies populations in Zhangjiakou (ZJ, EI populations), Chifeng (CF, EI populations), and Hinggan (HG, EI populations) as contemporary source areas actively supplying propagules to ecologically critical Xilingol grasslands (Wang et al., 2018). Therefore, surveillance and control resources should be prioritized in these hub regions. Specifically, monitoring efforts must focus on the transport corridors most likely to carry propagules—such as major livestock routes, agricultural machinery transit paths, and the trade flows of hay or crop residues—that connect these hubs to vulnerable regions (Rogers et al., 2019).

The bridgehead role of high-diversity populations, exemplified by Zhangjiakou, underscores a critical management principle: containing or eradicating well-established populations in key hubs is a preemptive strategy to prevent secondary invasions (van Boheemen et al., 2017). Investing in the control of these robust source populations before they spawn new satellite populations will likely yield greater long-term returns than solely managing already-dispersed, low-diversity front-line populations.

Furthermore, the prevalence of human-mediated jump dispersal, genetically inferred and field-validated via contaminated fodder, dictates that disrupting anthropogenic vectors should be a central component of management, rather than focusing solely on containing natural diffusion (Lin and Tan, 2007). Practical biosecurity measures should be implemented at key points along this pathway, including cleaning agricultural machinery moving from source regions, inspecting fodder sources at markets, and raising awareness among herders and farmers about the risks of using contaminated crop residues. By integrating genomic insights that pinpoint where and how invasions are spreading with landscape-level interventions at these precise points, this study enables a more proactive and precise defense of grassland ecosystems against *S. rostratum* and analogous invasive taxa (Pyšek et al., 2020).

### 4.5 Complementary insights from multiple analytical methods

To coherently integrate the findings from our primary analyses and address the nuanced relationships among them, we here explicitly discuss the complementary nature of the phylogenetic, ADMIXTURE, and PCA results. The apparent minor differences in the placement of certain populations (e.g., XLXH, Baicheng) stem from the distinct mathematical objectives and assumptions inherent to each method, yet together they reveal a consistent and complex population history. The Maximum Likelihood phylogenetic tree models bifurcating evolutionary relationships, depicting patterns of shared ancestry and divergence. In contrast, ADMIXTURE models ancestry proportions from predefined ancestral gene pools, where the chosen *K* value acts as a temporal filter—higher *K* values resolve recent, fine-scale structure, while lower *K* values capture deeper, shared ancestry. Principal Component Analysis (PCA), as a dimensionality reduction technique, extracts the primary axes of continuous genetic variation without *a priori* assumptions about discrete clusters or historical processes.

This methodological complementarity is best illustrated by the XLXH population. Its close phylogenetic branching and PCA proximity to Chifeng groups (CFBY, CFBZ, CFAL) suggest recent shared ancestry or gene flow. Its distinct ancestry profile under high-K ADMIXTURE (*K* = 7, 8, 17) reflects its subsequent genetic differentiation. The apparent contradiction is resolved by examining a lower *K* value (*K* = 4, **Supplementary Figure 5**), where XLXH shares a predominant ancestral component with the Chifeng populations, indicating a deep common gene pool. Thus, the tree and PCA capture recent genetic similarity, high-*K* ADMIXTURE characterizes recently diverged unique components, and low-*K* ADMIXTURE reveals deeper shared ancestral backgrounds.

The general agreement across all methods regarding the seven major genetic groups reinforces the biological reality of these core clusters. Their diverging signals for specific populations do not indicate data error but offer nuanced, hierarchical insights into admixture and historical layering. This integrative interpretation underscores that a multi-method approach is not merely redundant but essential for reconstructing complex invasion histories involving successive founder events, admixture, and continuous dispersal.

### 4.6 Limitations and future perspectives

While our genomic data robustly delineate dispersal routes and source-sink dynamics among Chinese populations, some limitations should be acknowledged. First, the absence of genomic data from native-range populations (Mexico and the USA) precludes a definitive reconstruction of the initial introduction(s) into China and limits our ability to conclusively quantify the relative contribution of direct introductions versus secondary spread within the country. However, the strong genetic connectivity and admixture signals observed among domestic populations, coupled with the invasion chronology, make ongoing bridgehead dynamics a parsimonious explanation for the contemporary expansion. Second, our study identified and verified one specific anthropogenic vector (contaminated fodder trade) through a targeted survey. While this provides crucial mechanistic validation, the dispersal of invasive plants often involves multiple pathways. We did not systematically quantify the relative contribution of other potential vectors such as agricultural machinery transport, seed contamination, or vehicular dispersal, which likely also facilitate jump dispersal in this system. Future research should integrate large-scale surveillance of agricultural and transportation networks with genomic assignment methods to quantify the prevalence and impact of different human-mediated pathways. Such work will further refine risk assessment and target the most significant dispersal corridors for management intervention.

## 5 Conclusion

This study integrated population genomics with invasion history to test hypotheses on the human-mediated dispersal of *S. rostratum* in China. We found substantial and convergent support for both hypotheses: newly invaded populations are genetically admixed from multiple established sources (H1), and asymmetric gene flow identifies early-invaded populations in Zhangjiakou (ZJ), Chifeng (CF), and Hinggan (HG) as key anthropogenic dispersal hubs (H2). Although a weak isolation-by-distance pattern reflects limited natural diffusion, the profound genetic-geographic decoupling—combined with field-verified evidence of contaminated fodder trade—confirms that human-mediated jump dispersal is the dominant mechanism driving current expansion, characteristic of a bridgehead dynamic. Consequently, effective management must shift from broadly containing spread to precisely targeting these source hubs and the human-mediated corridors (especially agricultural supply chains) that link them to vulnerable grasslands such as Xilingol. Our findings thus provide a genetic framework for developing targeted, preemptive strategies to enhance grassland biosecurity against *S. rostratum* and analogous invasive species.

## Data Availability

The data presented in the study are deposited in the GenBank repository, accession numbers PRJNA1176205.
