# Supplementary material for: Population genomics reveals genetic structure and dispersal sources of the invasive herb Solanum rostratum in northern China
Source: Front Plant Sci. 2026 Mar 20;17:1688630. doi: 10.3389/fpls.2026.1688630 (PMC13047094; doi:10.3389/fpls.2026.1688630)

Supplementary Material

1. **Supplementary Table S1. Sampled population information and population-level genetic diversity indices for *Solanum rostratum* in China**

Detailed data are provided in a separate file: Supplementary Table S1.xlsx.

1. **Supplementary Table S2. Genome sequencing statistics for** ***Solanum rostratum* samples from China**

Detailed data are provided in a separate file: Supplementary Table S2.xlsx.

1. **Supplementary Table S3. Pairwise *Fst* values among the 30** ***Solanum. rostratum* populations in China**

Detailed data are provided in a separate file: Supplementary Table S3.xlsx.

1. **Supplementary Table S4. Pairwise geographical distances (km) among the 30 *Solanum rostratum* populations in China**

Detailed data are provided in a separate file: Supplementary Table S4.xlsx.

1. **Supplementary Table S5. Estimated gene flow (migration rates) among the 30 *Solanum rostratum* populations in China**

Detailed data are provided in a separate file: Supplementary Table S5.xlsx.

1. **Supplementary Table S6.** **Summary of genetic diversity indices for *Solanum rostratum* across different invasion stages (Early-invaded, Mid-invaded, Newly-invaded)**

Detailed data are provided in a separate file: Supplementary Table S6.xlsx.

1. **Supplementary Table S7. Summary of *Solanum rostratum* populations sampled in this study, categorized by invasion stage with corresponding codes (EI, MI, NI)**

The table lists all populations with their full names, abbreviations, invasion stage classification (EI, MI, NI), and corresponding temporal codes used in the manuscript.

Detailed data are provided in a separate file: Supplementary Table S7.xlsx.


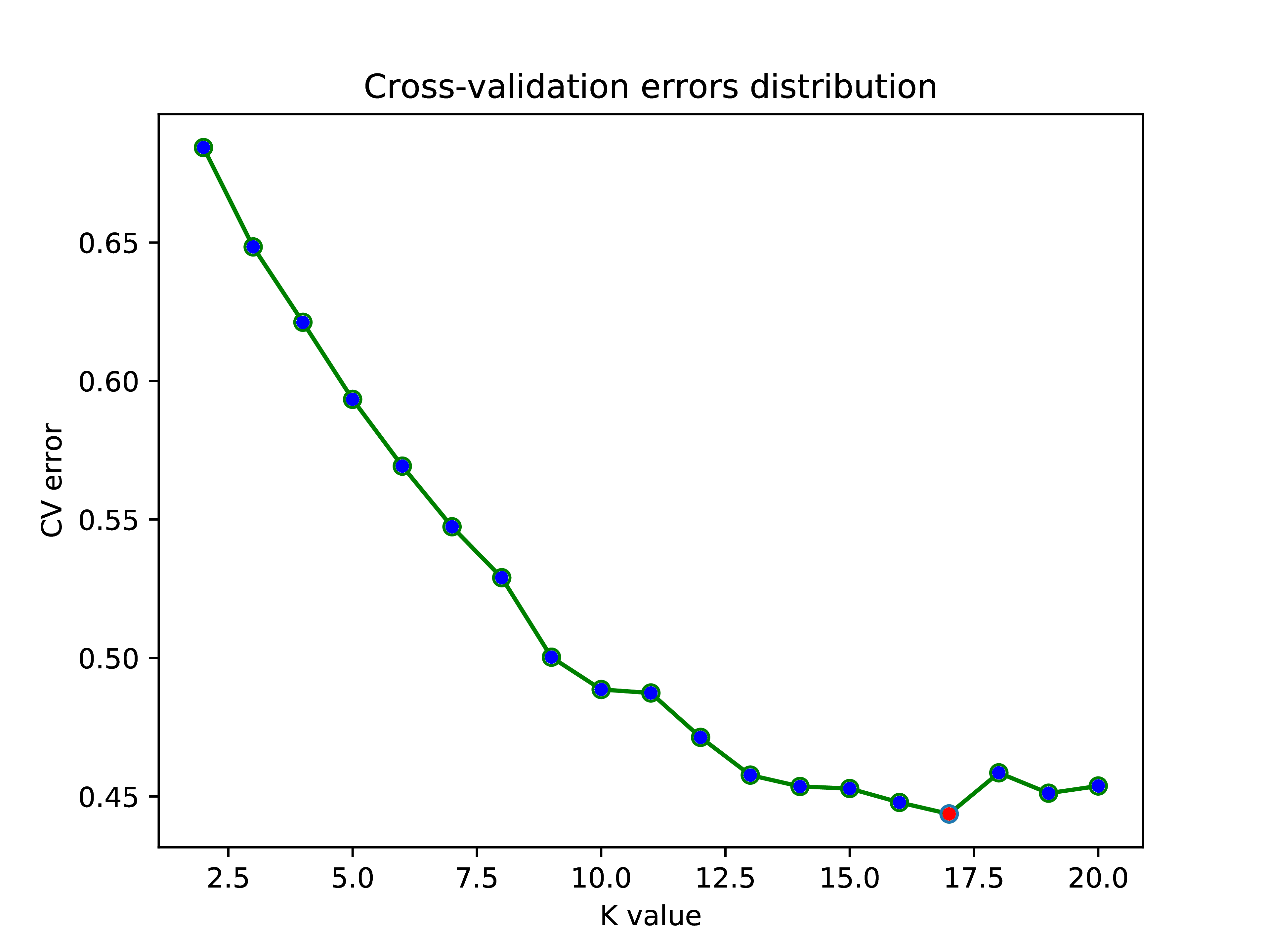


1. **Supplementary Figure S1. CV error plot from ADMIXTURE analysis.**

The plot shows the cross-validation (CV) error for different numbers of ancestral clusters (*K*), with the minimum error indicating the statistically optimal *K* value.


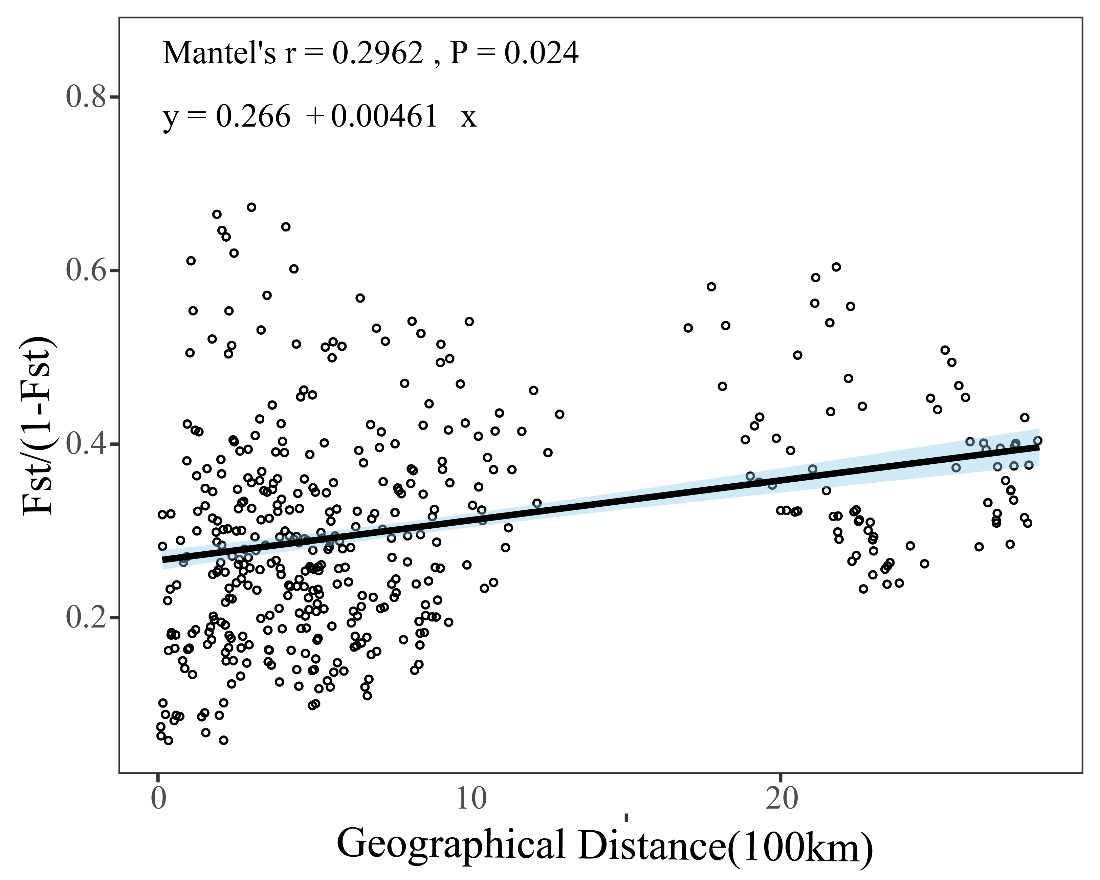


1. **Supplementary Figure S2. Isolation-by-distance pattern for *Solanum rostratum* populations in China.**

Genetic distance (*F*<sub>ST</sub>) is plotted against geographical distance (in 100 km intervals). The blue dashed line represents the linear regression fit (Mantel test: *r* = 0.29).


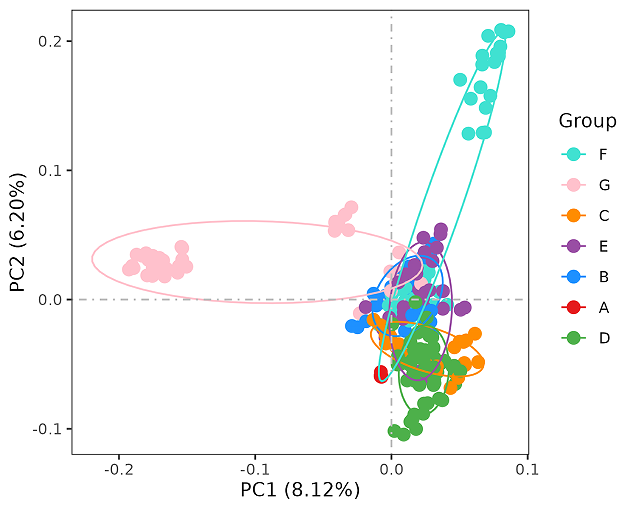


1. **Supplementary Figure S3. Principal Component Analysis (PCA) of *Solanum rostratum* individuals.**

The scatterplot shows the first two principal components, with individuals colored according to the seven genetic subgroups (gene pools) identified in the study. The percentage of variance explained by each principal component is indicated on the axes.

1. **Supplementary Figure S4. Comparative validation of population genetic analyses using independent SNP calling pipelines (Stacks v2.3).**

This section provides a self-contained validation report to assess the robustness of the main findings presented in the manuscript (which are based on GATK-derived SNPs). Below, we present the key population genetic analyses repeated using the Stacks pipeline. For direct visual comparison, the key results from the Stacks pipeline are presented side-by-side with the corresponding GATK-based results (reproduced from the main text figures). The high concordance between the two independent analyses confirms that the core conclusions regarding genetic structure, phylogeny, and diversity trends are not dependent on the choice of SNP calling methodology.

*Genetic Structure, Maximum Likelihood (ML) Tree and Spatial genetic structure*

Cross-validation analysis of the STACKS-derived SNP data indicated K=19 as the optimal value with the lowest cross-validation error, which is largely consistent with the optimal K=17 obtained from the GATK analysis. Following established population clustering patterns, we further examined the genetic structure at K=7, K=8 and K=17. The results at these levels revealed a high degree of concordance between the two datasets. Specifically, at K=7, populations from Chifeng and Baicheng were clearly distinguished as distinct clusters in both analyses. At K=8, the XLXH population was distinctly separated. Furthermore, the topology of the Maximum Likelihood (ML) phylogenetic trees was identical between the two software. Analyses of ancestral composition using spatial data from both software show largely consistent results, with only minor discrepancies.

*Genetic Diversity*

A direct comparison of genetic diversity indices calculated from the two SNP sets revealed minor quantitative differences. However, the key qualitative pattern was consistent: all major indices, including effective population size (*Ne*), observed heterozygosity (*Ho*), expected heterozygosity (*He*), inbreeding coefficient (*Fis*), and nucleotide diversity (*π*), showed a progressive decline across the defined invasion stages.


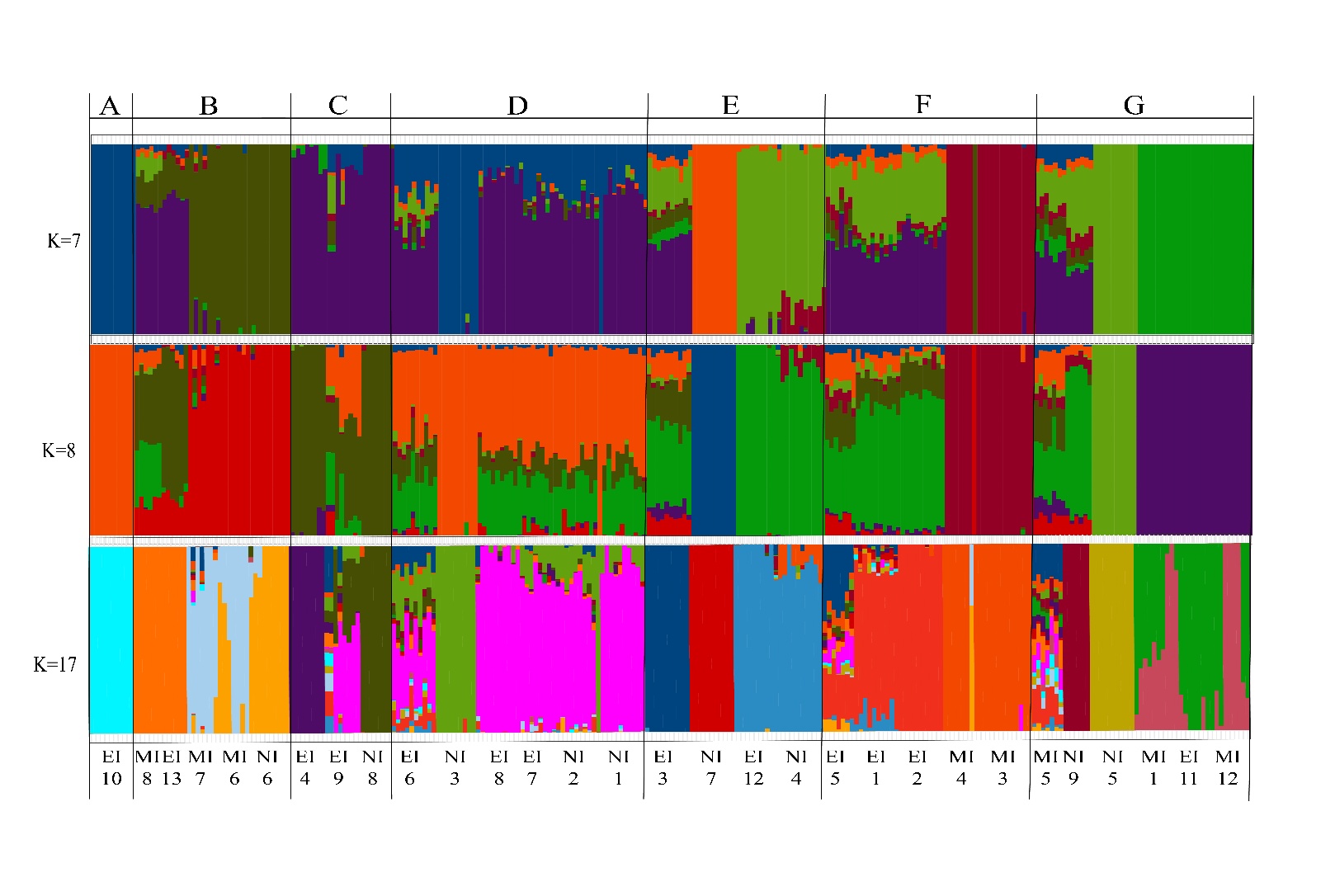

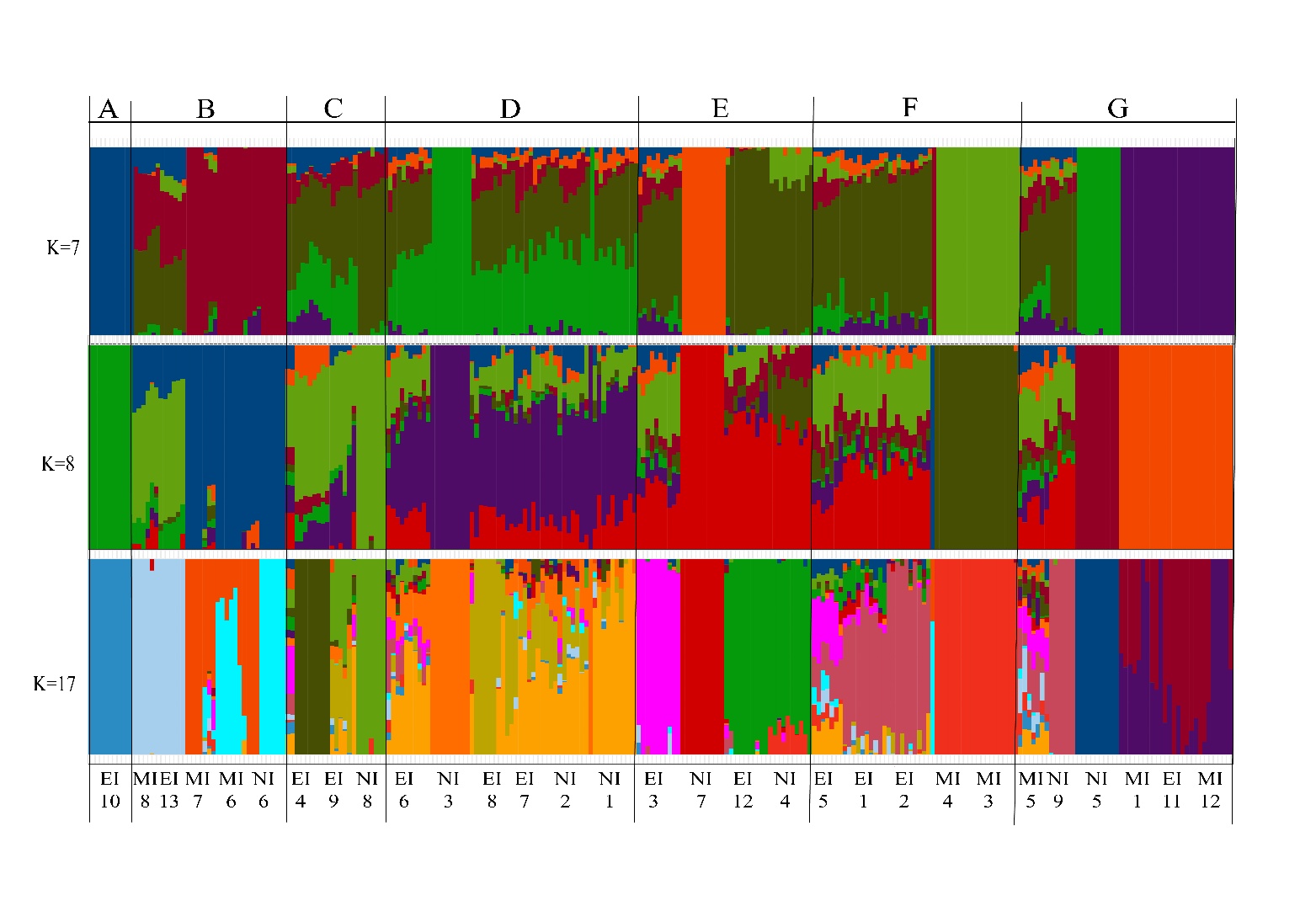


**Figure S4A. Genetic structure inference.**

(*Left*) ADMIXTURE analysis based on SNPs called using Stacks (K=7 and K=8). (*Right*) Corresponding results based on GATK (reproduced from main text Figures 2 for comparison). Both analyses reveal nearly ientical ancestry proportions and clustering patterns. Key populations (e.g., Chifeng and Baicheng at K=7, XLXH at K=8) are consistently distinguished as distinct genetic groups in both pipelines.


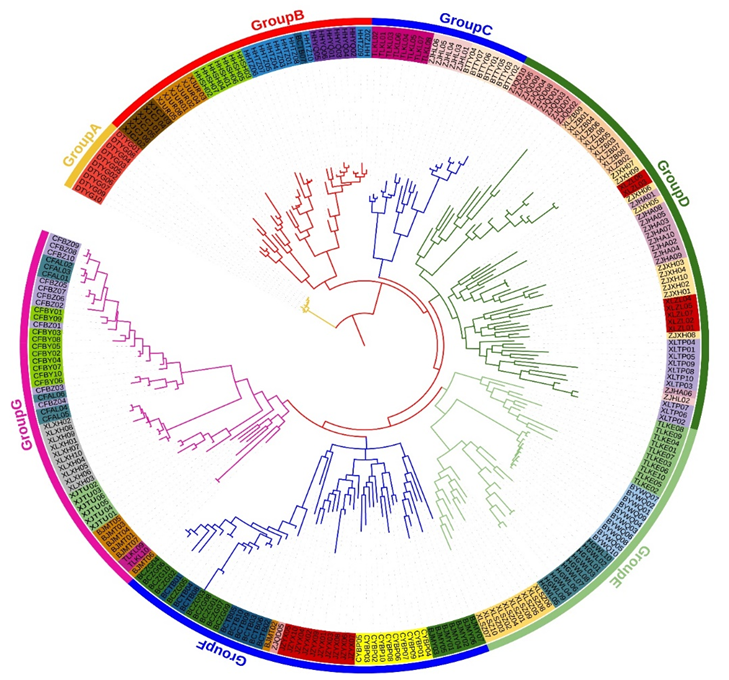

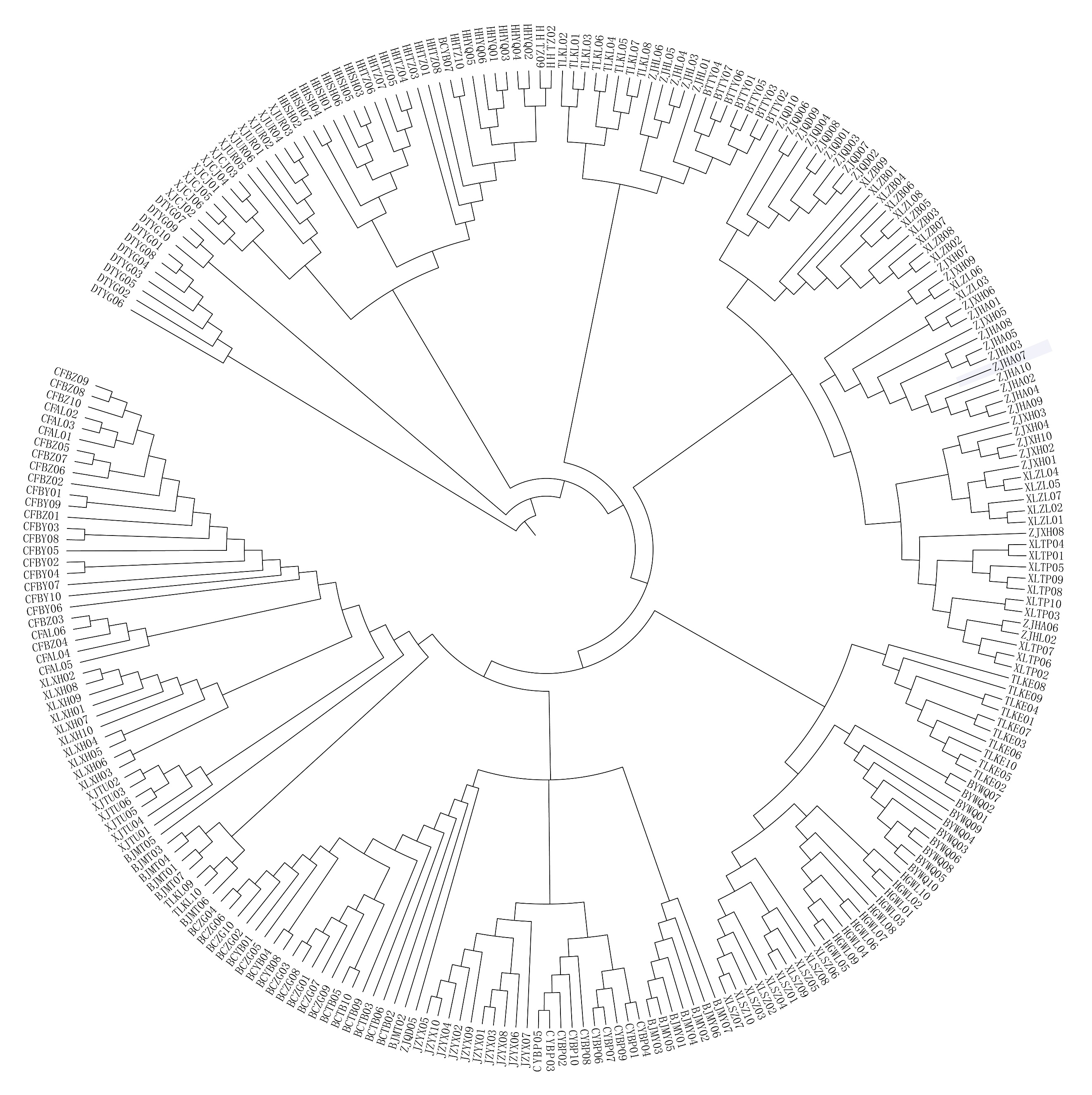


**Figure S4B. Phylogenetic relationships.**

(*Left*) Maximum Likelihood (ML) tree based on SNPs called using Stacks. (*Right*) Corresponding ML tree based on GATK (reproduced from main text Figure 3 for comparison). The tree topologies are identical, supporting the same seven monophyletic groups corresponding to the major gene pools identified in the study.


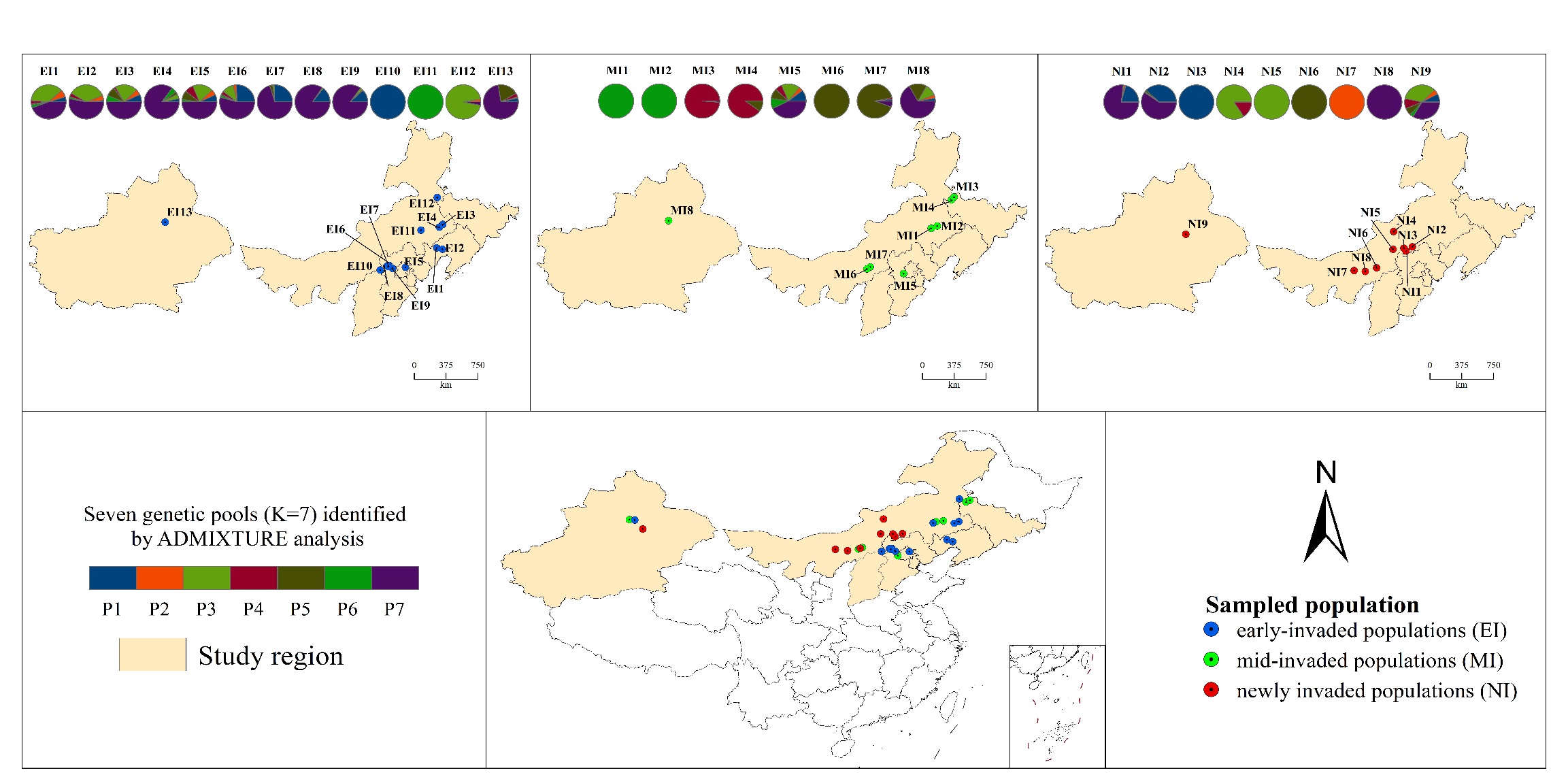

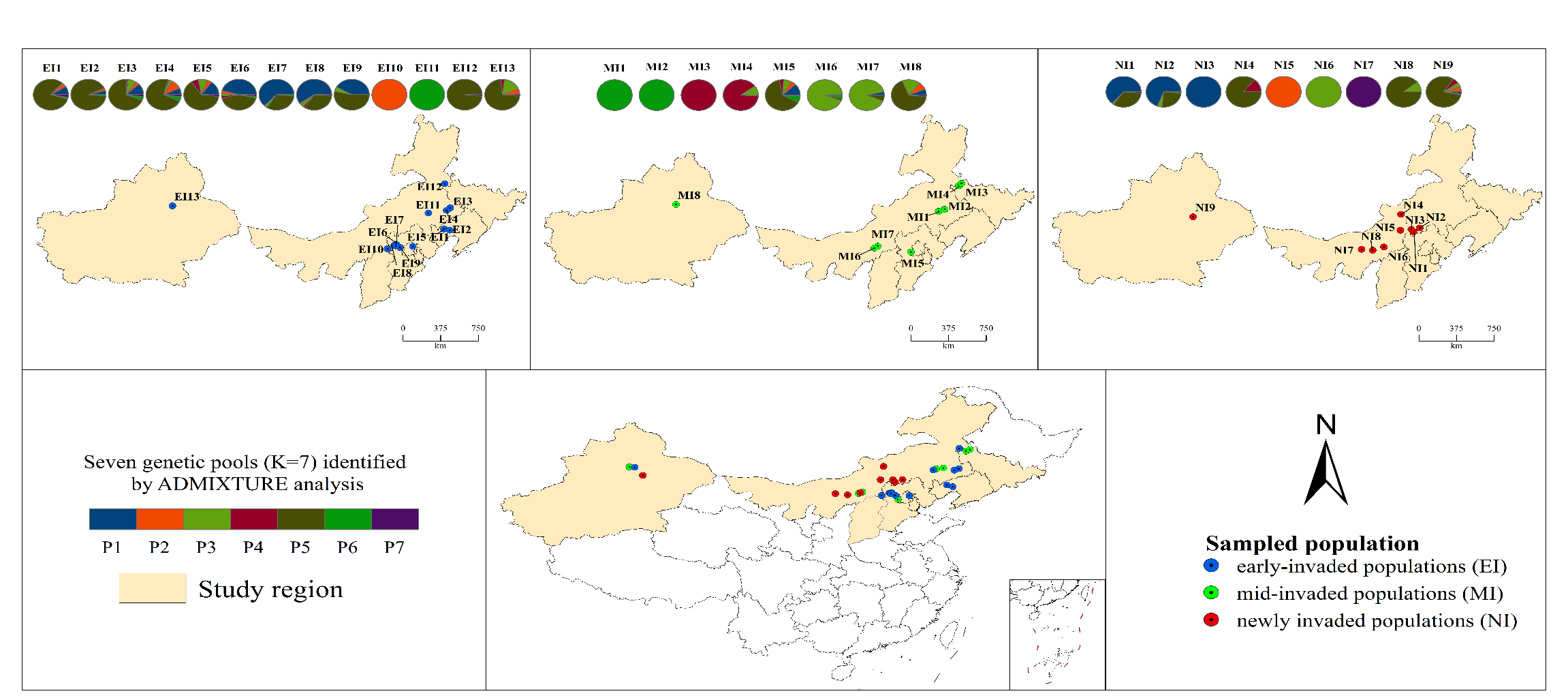


**Figure S4C. Spatial genetic structure of *Solanum rostratum* populations across China.**

(Top) Proportional ancestry across sampling locations, inferred using ADMIXTURE (K = 7) based on SNP data from Stacks.

(Bottom) Spatial genetic structure corresponding to the above, analyzed via GATK.


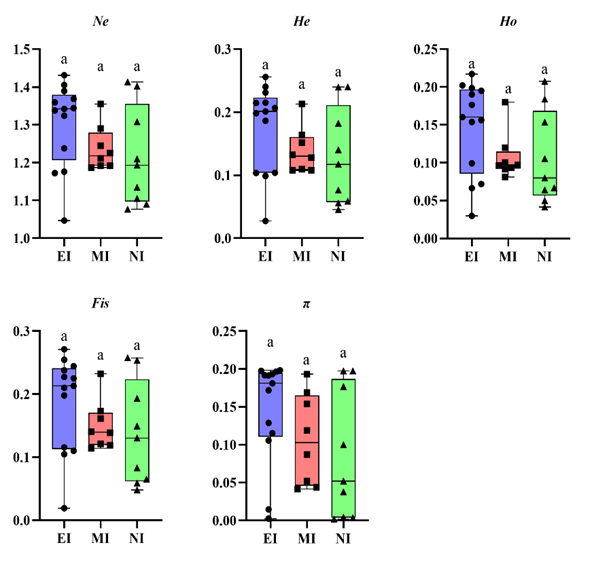

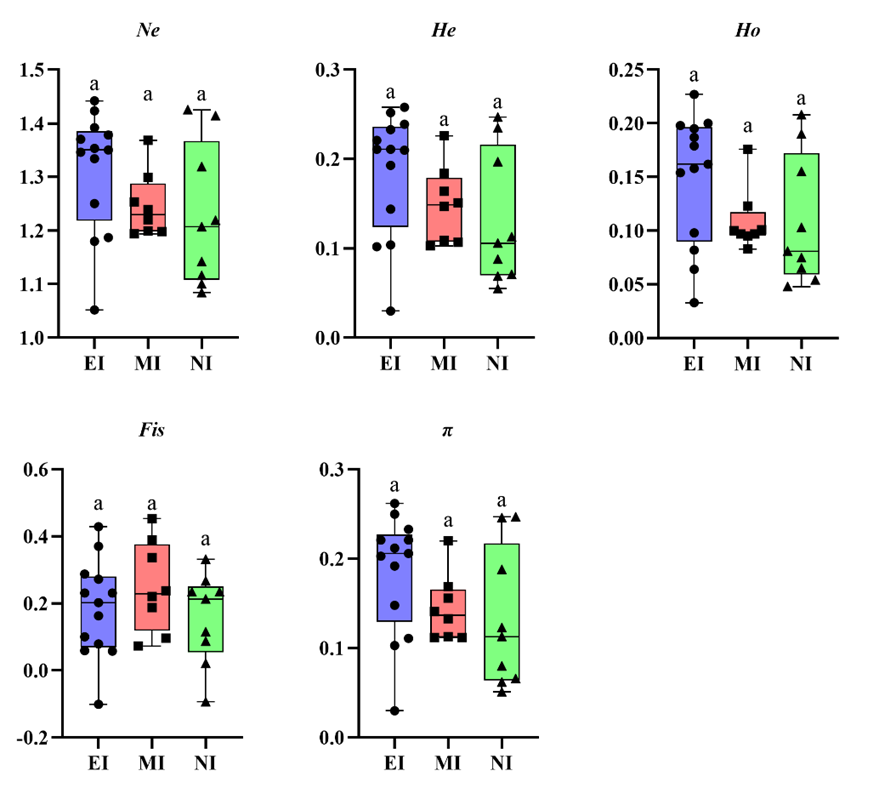


**Figure S4D. Trends in genetic diversity across invasion stages.**

(*Left*) Boxplots of genetic diversity indices (*Ne*, *Ho*, *He*, *Fis*, π) calculated from Stacks-derived SNPs. (*Right*) Corresponding boxplots from GATK-derived SNPs (reproduced from main text Figure 7 for comparison).

Both datasets exhibit the same qualitative and quantitative trend: a progressive decline in genetic diversity from early- (EI) to newly invaded (NI) populations, validating the pattern reported in the main text.

**Conclusion of Comparative Validation:** The independent analyses conducted with the Stacks pipeline yield results that are highly congruent with those obtained from GATK across all critical dimensions: population structure assignment, phylogenetic topology, and trends in genetic diversity. This consistency demonstrates that the core biological inferences and conclusions of our study are robust and not an artifact of the specific SNP calling algorithm employed.

1. **Figure S5. Genetic structure inferred by ADMIXTURE at K=4.**

The seven genetic clusters (G1–G7) correspond precisely to the seven major groups identified in the Maximum Likelihood phylogenetic tree (Figure 2).


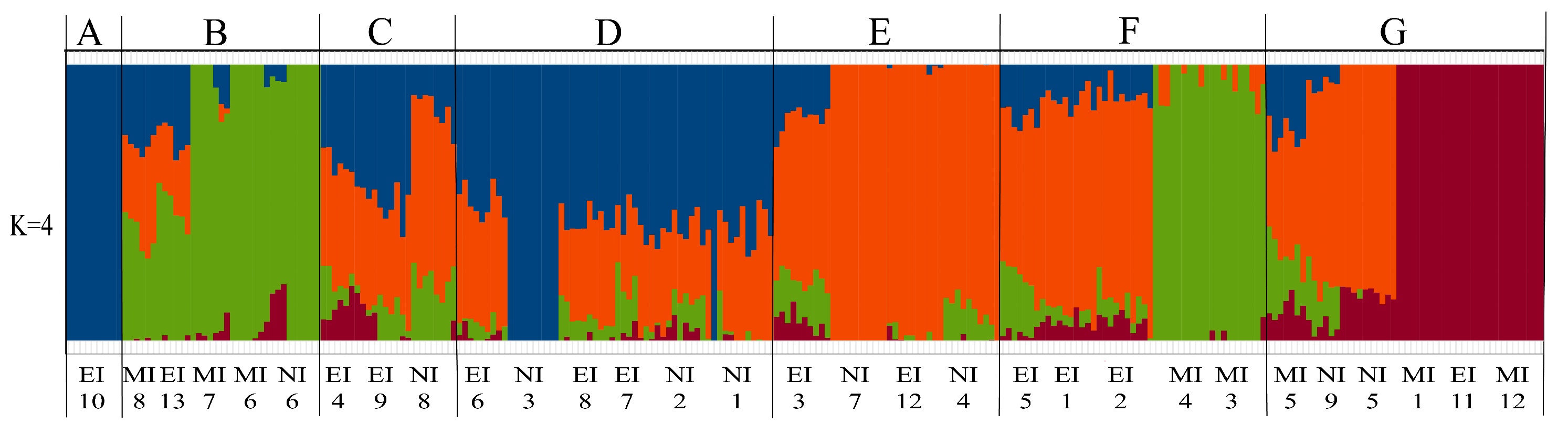

Supplement: Supplementary file 1 [file DataSheet1.zip › Supplementary Material-final/Supplementary_Material1.docx]
